# Real-World Outcomes of Ivacaftor Treatment in People with Cystic Fibrosis: A Systematic Review

**DOI:** 10.3390/jcm10071527

**Published:** 2021-04-06

**Authors:** Jamie Duckers, Beth Lesher, Teja Thorat, Eleanor Lucas, Lisa J. McGarry, Keval Chandarana, Fosca De Iorio

**Affiliations:** 1 All Wales Adult Cystic Fibrosis Centre, University Hospital Llandough, Cardiff CF64 2XX, UK; 2 Pharmerit—An OPEN Health Company, 4350 East-West Highway, Suite 1100, Bethesda, MD 20814, USA; blesher@pharmerit.com (B.L.); elucas@pharmerit.com (E.L.); 3 Vertex Pharmaceuticals Incorporated, Boston, MA 02210, USA; Teja_Thorat@vrtx.com (T.T.); Lisa_McGarry@vrtx.com (L.J.M.); Keval_Chandarana@vrtx.com (K.C.); 4 Vertex Pharmaceuticals (Europe) Limited, London W2 6BD, UK; Fosca_DeIorio@vrtx.com

**Keywords:** cystic fibrosis, cystic fibrosis transmembrane conductance regulator (CFTR), ivacaftor, real-world evidence, systematic literature review

## Abstract

Cystic fibrosis (CF) is a rare, progressive, multi-organ genetic disease. Ivacaftor, a small-molecule CF transmembrane conductance regulator modulator, was the first medication to treat the underlying cause of CF. Since its approval, real-world clinical experience on the use of ivacaftor has been documented in large registries and smaller studies. Here, we systematically review data from real-world observational studies of ivacaftor treatment in people with CF (pwCF). Searches of MEDLINE and Embase identified 368 publications reporting real-world studies that enrolled six or more pwCF treated with ivacaftor published between January 2012 and September 2019. Overall, 75 publications providing data from 57 unique studies met inclusion criteria and were reviewed. Studies reporting within-group change for pwCF treated with ivacaftor consistently showed improvements in lung function, nutritional parameters, and patient-reported respiratory and sino-nasal symptoms. Benefits were evident as early as 1 month following ivacaftor initiation and were sustained over long-term follow-up. Decreases in pulmonary exacerbations, *Pseudomonas aeruginosa* prevalence, and healthcare resource utilization also were reported for up to 66 months following ivacaftor initiation. In studies comparing ivacaftor treatment to modulator untreated comparator groups, clinical benefits similarly were reported as were decreases in mortality, organ-transplantation, and CF-related complications. The safety profile of ivacaftor observed in these real-world studies was consistent with the well-established safety profile based on clinical trial data. Our systematic review of real-world studies shows ivacaftor treatment in pwCF results in highly consistent and sustained clinical benefit in both pulmonary and non-pulmonary outcomes across various geographies, study designs, patient characteristics, and follow-up durations, confirming and expanding upon evidence from clinical trials.

## 1. Introduction

Cystic fibrosis (CF) is a rare, multi-organ genetic disease that affects an estimated 70,000–100,000 people worldwide [1]. CF results from mutations in the CF transmembrane conductance regulator (*CFTR*) gene, which impact on CFTR protein expression and/or function at the epithelial cell surface [2]. This leads to defective transport of chloride and sodium ions across the epithelial cell membrane of multiple organs, including the lungs, pancreas, gallbladder, intestine, and reproductive system [3].

Respiratory failure is the primary cause of death for people with CF (pwCF) [3]. Generally, lung function, as assessed by percent predicted forced expiratory volume in 1 s (ppFEV_1_), declines by 1–3 percentage points annually, with the steepest declines occurring in early adulthood [4]. Lung function is also negatively affected by pulmonary exacerbations (PEx), an acute worsening of signs and symptoms that may require hospitalization and treatment with intravenous (IV) antibiotics [5,6]. Following a PEx, many pwCF do not recover baseline lung function and experience sustained decreases in ppFEV_1_ [6]. CF is also associated with extra-pulmonary complications. Significant exocrine pancreatic damage is thought to occur starting in utero, resulting in insufficient production of pancreatic enzymes for digestion, often from birth. In combination with gastrointestinal dysfunction, this can lead to poor nutrition and growth [7,8,9]. Moreover, CF is associated with damage to the endocrine pancreas over time, leading to the development of CF-related diabetes (CFRD) in some pwCF [5,10]. These comorbidities result in high levels of healthcare utilization and have been linked to morbidity and mortality [9].

A model examining predictors of 5-year survival in pwCF using data from the US CF Patient Foundation Registry showed higher ppFEV_1_ is associated with increased 5-year survival [11]. This model also identified pancreatic sufficiency, higher weight-for-age *z*-score, and *Staphylococcus aureus* infection as being associated with improved survival, whereas presence of *Burkholderia cepacia* infection, diabetes mellitus, female sex, higher number of PEx, and increased age were associated with decreased survival [11,12].

Historically, CF treatment focused on addressing clinical symptoms, maintaining lung function, improving nutritional status, and managing pancreatic insufficiency, organ damage, and disease sequelae, such as CFRD [13]. Improvements in the standard of care have prolonged survival for pwCF; however, the median age at death was 30.8 years in 2018 in the US [13] and 29.0 years in 2017 in Europe [14]. The recent development of CFTR modulators that address the underlying cause of CF has the potential to change the disease trajectory. Ivacaftor (Kalydeco^®^, Vertex Pharmaceuticals Incorporated, Boston, MA, USA), a CFTR potentiator, was the first approved CFTR modulator therapy for pwCF aged ≥6 years with a *G551D CFTR* mutation [15,16]. Following initial approval, ivacaftor has been granted a series of label expansions by the US Food and Drug Administration (FDA) and the European Medicines Agency to include additional *CFTR* mutations in pwCF as young as 4 months of age [17,18]. Ivacaftor clinical trials that enrolled pwCF with *G551D* mutations aged 6–11 years (ENVISION) and ≥12 years (STRIVE) demonstrated significant improvements in pulmonary, nutritional, and patient-reported outcomes (PROs) over 24 and 48 weeks of treatment [15,16]. Ivacaftor was generally safe and well tolerated, with the majority of observed adverse events consistent with CF disease manifestations [15,16]. Improvements observed in pwCF treated with ivacaftor in these trials were maintained during a 96-week open-label extension study in pwCF aged ≥6 years (PERSIST) [19]. Clinical trials in pwCF aged 2–5 years (KIWI) and 4 to <24 months (ARRIVAL) showed similar ivacaftor pharmacokinetics to those reported in older children and adults, with acceptable safety profiles [20,21,22,23]. Improvements in clinical outcomes with ivacaftor treatment also were seen in clinical trials enrolling pwCF with non-*G551D* gating mutations (KONNECTION), the *R117H* mutation (KONDUCT), and other mutations [24,25,26].

Clinical trial data provide important evidence of safety and efficacy, but there is considerable interest in understanding real-world outcomes associated with ivacaftor in routine clinical practice. Here, we systematically review and qualitatively synthesize real-world evidence available for pwCF treated with ivacaftor.

## 2. Methods

We performed a literature review based on a predefined search and selection protocol (Table S1). Search criteria included: (i) studies published as journal articles or conference abstracts between 1 January 2012 and 4 September 2019 in MEDLINE or Embase (searched via ProQuest), and (ii) studies written in the English language. The start date of 1 January 2012 was chosen to correspond with the FDA approval month for ivacaftor.

Studies included in the review enrolled at least six pwCF of any age and from any country that reported outcomes for pwCF treated with ivacaftor as monotherapy (i.e., not in combination with other CFTR modulators). Clinical case reports as well as interventional (randomized or non-randomized), animal, and preclinical studies were excluded.

Key outcomes were prespecified by clinical experts and included those related to lung function (e.g., ppFEV_1_), PEx, nutritional parameters (e.g., body mass index [BMI], weight, fat-free mass, weight *z*-score, height *z*-score, and weight-for-age), endocrine and exocrine pancreatic functions (e.g., CFRD, pancreatic insufficiency), healthcare utilization (e.g., hospitalizations, antibiotic utilization and duration), lung microbiome, PROs (e.g., Cystic Fibrosis Questionnaire-Revised (CFQ-R) respiratory symptom scale, Sino-Nasal Outcome Test (SNOT), and physical function tests, such as 6-min walk test and shuttle walk test), safety, CF-related complications, organ transplantation, and mortality.

Two reviewers independently screened all study titles and abstracts to identify citations for full-text review. A third independent reviewer resolved discrepancies. The review followed Preferred Reporting Items for Systematic Reviews and Meta-Analyses guidelines [27]. Where multiple publications reported the same outcomes from the same study population at the same time point, those from the most recent publication were retained.

Data were extracted and are reported as set forth in the publications, except as noted. For studies including both ivacaftor-treated and modulator untreated comparison groups, data were extracted from both groups as reported in the publications, either as change in mean values from baseline to follow-up for each group (“change scores”) or as difference in the change scores at follow-up. “Baseline” is defined as per the publication, as either cross-sectional values recorded at the time of enrollment or ivacaftor treatment initiation, or as values collected over a predefined time period (e.g., 12 months) prior to ivacaftor initiation. In studies including a comparator group, the modulator untreated comparator cohort comprised pwCF treated with symptomatic CF treatments, but not including CFTR modulators. For studies without a comparison group, the impact of ivacaftor treatment is reported as within-group change scores in the ivacaftor-treated cohort from baseline. To facilitate summarizing ppFEV_1_, BMI, and weight graphically across studies, change scores (or absolute change) from baseline in the ivacaftor-treated cohort were calculated from abstracted data when not reported in the publication.

## 3. Results

### 3.1. Demographics, Patient Populations, and Study Designs

**Figure 1:**
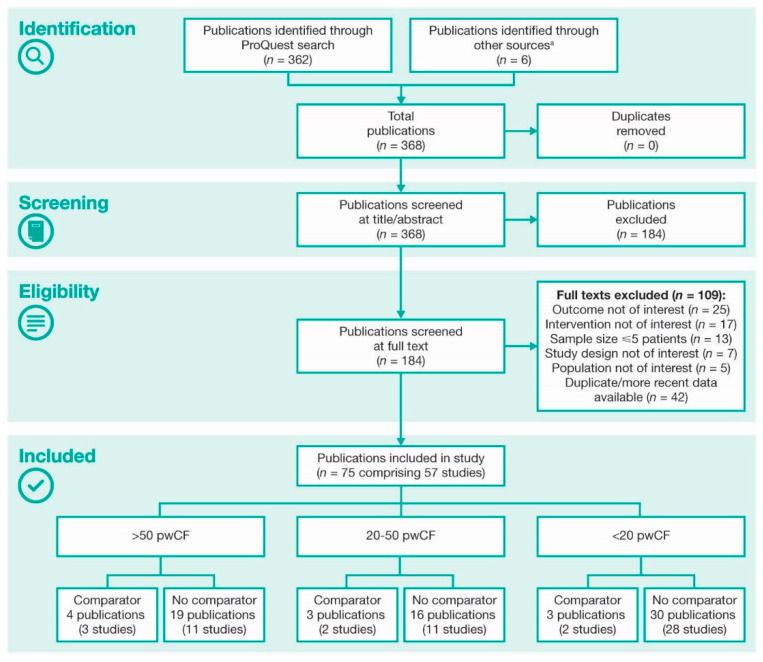
Identification of Publications According to PRISMA Guidelines. ^a^ Identified via European Cystic Fibrosis Society Conference summaries. PRISMA: Preferred Reporting Items for Systematic Reviews and Meta-Analyses; pwCF: people with cystic fibrosis.

**Figure 2:**
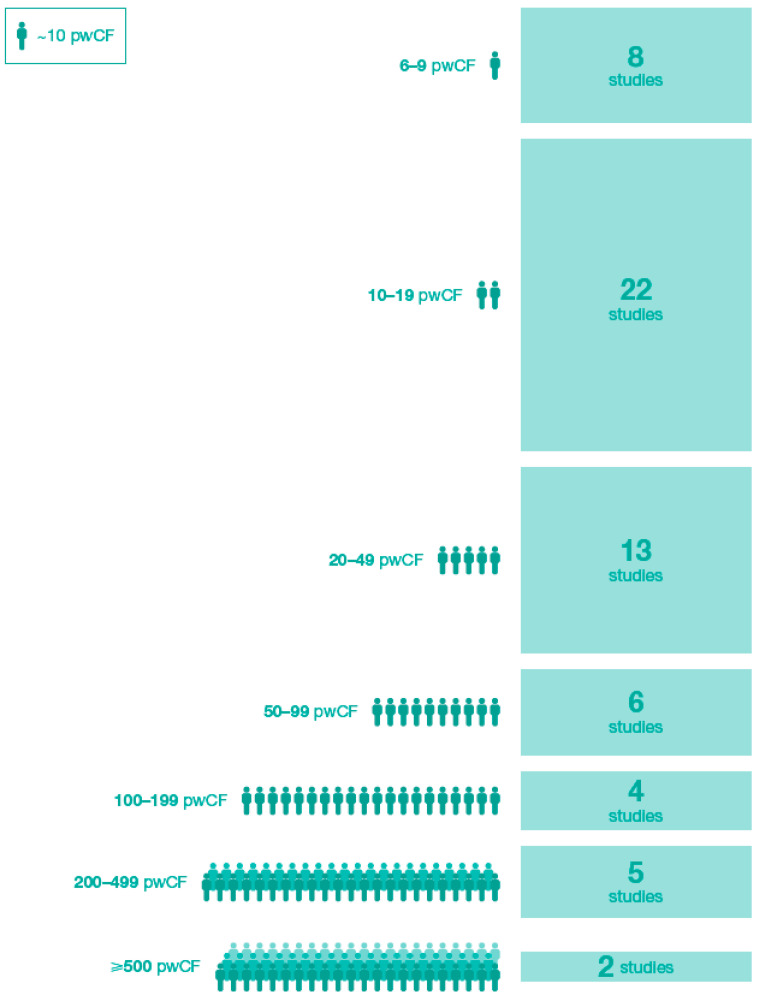
Real-world studies by ivacaftor-treated cohort size. Note: Studies do not sum to 57 because the Long-Term Safety Study reported results separately for US and UK cohorts treated with ivacaftor in cross-sectional [29] and disease progression analyses [30]. pwCF: people with cystic fibrosis.

Three-hundred and sixty-eight publications were identified and the abstracts reviewed, of which 184 publications were selected for full-text review (Figure 1). Seventy-five publications (28 manuscripts; 47 conference abstracts), comprising 57 unique studies, met inclusion criteria for analyses (Table 1; Table S2). The ivacaftor-treated cohorts in these studies ranged in size from six to 1256 pwCF (Figure 2); >50% of studies included <20 pwCF.

Fifteen (26%) studies were from the UK [29,30,31,55,58,59,60,69,70,74,78,82,83,87,90,91,93,100], 13 (23%) from the US [29,30,35,38,41,42,43,44,45,46,47,48,49,50,56,67,68,72,75,98,99], 17 (30%) from non-UK European countries [34,51,52,53,54,61,62,63,64,65,66,71,77,79,84,86,94,95,96,97,102], three (5%) from Canada [80,81,88,92], two (4%) from Australia [36,37,101], one (2%) from Israel [76], one (2%) from the United Arab Emirates [57], and one (2%) from Oman [73] (Table 1). Five (9%) studies were multinational [28,32,33,39,40,89]. Thirteen (23%) studies had follow-up times of <12 months [38,69,72,78,79,82,84,86,87,88,89,94,102], 26 (46%) had follow-up times of 12–23 months [28,34,37,39,40,48,49,50,52,53,56,57,59,62,66,67,68,71,73,75,76,81,83,91,95,96,97,98,99], 14 (25%) had follow-up times of 24–59 months [31,33,51,54,55,61,70,74,77,85,90,92,100,101], and three (5%) studies had follow-up times of ≥60 months [30,41,93]; one (2%) study did not report length of follow-up [35].

Thirty-one (54%) studies included both adult and pediatric populations [28,29,30,38,39,40,41,42,43,44,45,46,48,49,50,51,52,53,54,56,62,66,68,69,72,75,76,77,79,80,81,86,87,88,89,90,92,96,98,99,100], whereas 16 (28%) studies included only adult populations [32,33,36,37,58,60,61,70,71,74,82,83,84,85,94,95,97,101,102] and five (9%) studies included only pediatric populations [34,35,41,42,51,57,67,70,93]. There were five (18%) studies that did not report the age of the included population [31,55,73,78,91]. Thirty-nine (68%) studies were restricted to pwCF who had a *CFTR* gating mutation [28,31,32,33,36,37,39,40,43,44,45,47,49,51,52,53,54,57,58,59,60,61,62,63,64,65,66,69,70,72,74,76,77,78,79,80,81,83,84,85,86,87,88,89,90,92,93,96,97,98,99,101,102]; among these, 20 (51%) included only pwCF with a *G551D* mutation [28,31,32,33,36,37,49,51,54,58,59,60,63,64,65,66,74,77,78,79,83,84,85,87,90,98,99,101,102]. Six (11%) studies included pwCF exclusively with severe lung disease (i.e., ppFEV_1_ < 40%) [32,33,36,37,71,84,94,96].

A total of seven (12%) studies compared outcomes in ivacaftor-and non-ivacaftor-(i.e., modulator untreated comparator) treated cohorts [28,29,30,31,32,33,34,35,36,37]. Of these, two studies were limited to pwCF with severe lung disease receiving ivacaftor through a compassionate-use program and were compared to matched modulator untreated controls [32,33,37]. Of particular importance is the post-authorization surveillance study using data from the US and the UK CF registries (henceforth referred to as LTSS). The study consisted of ivacaftor-treated pwCF matched to comparators not eligible for ivacaftor based on patient characteristics including CFTR genotype severity [29,30]. The disease progression cohort of the LTSS consisted of pwCF who were prescribed ivacaftor and followed prospectively for up to 5 years in the US and up to 4 years in the UK [30]. A separate publication from the same study summarizes the results of cross-sectional analyses conducted using 2014 data from the US and UK CF registries. The mean length of ivacaftor exposure in the cross-sectional analyses of the LTSS was 2.0 years in the US cohort and 1.3 years in the UK cohort [29]. The majority of studies that included a modulator untreated comparator cohort either ensured comparability by matching ivacaftor-treated and untreated comparator groups based on patient characteristics including disease severity, age, and sex or included pwCF not treated with ivacaftor matched for age and sex. For instance, in the LTSS study pwCF with a *G551D* mutation (Class III mutations) were matched to pwCF who were homozygous for *F508del* mutation (Class II mutation) based on published evidence documenting the similarity in disease characteristics and disease progression between the two mutation groups [103,104].

The majority of identified studies (*n* = 50) did not have a modulator untreated comparator cohort. The follow-up period for those studies ranged from 1–66 months. Of these, 17 studies had baseline values collected during a period before ivacaftor therapy, whereas 33 studies followed pwCF after starting ivacaftor therapy, with baseline values reported at the time of enrollment or ivacaftor treatment initiation. Of the 50 studies, 11 were large multicenter registry or database studies that included data for ≥50 pwCF [39,40,41,42,43,44,45,46,47,52,53].

### 3.2. Lung Function and Pulmonary Exacerbations

#### 3.2.1. ppFEV_1_

**Figure 3:**
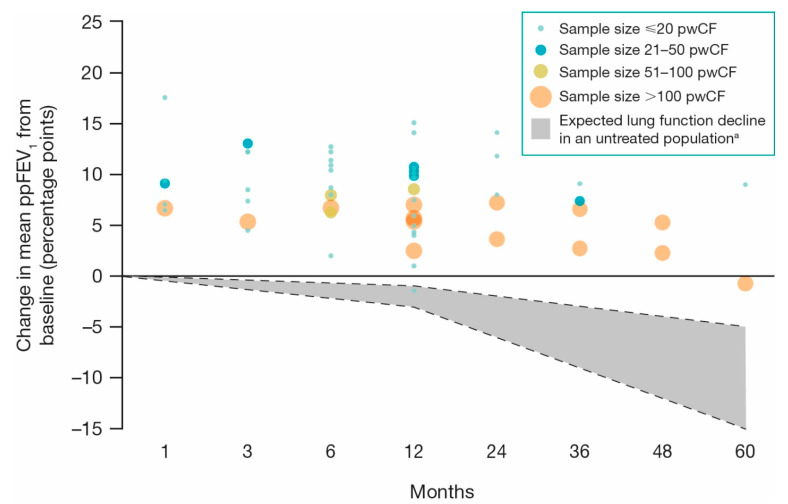
Change in mean ppFEV_1_ with ivacaftor treatment (31 studies) [30,40,41,42,44,45,47,49,53,54,57,58,62,66,72,73,74,75,76,77,78,82,83,86,87,91,92,93,95,98,99]. Note: Figure only includes studies reporting absolute change from baseline within the ivacaftor-treated cohort in ppFEV_1_ at 1, 3, 6, 12, 24, 36, 48, and/or 60 month time points following ivacaftor treatment. Excludes ppFEV_1_ for pwCF with severe lung disease (*n* = 3) [71,94,96]. ^a^ The grey area represents the range of ppFEV_1_ decline in pwCF treated with standard therapies not including CFTR modulators, being between 1–3 percentage points annually, which, when extrapolated to 60 months, is in the range of 5–15 percentage points [4]. CFTR: cystic fibrosis transmembrane conductance regulator; ppFEV_1_: percent predicted forced expiratory volume in 1 s; pwCF: people with cystic fibrosis.

Improved lung function, as indicated by increases in ppFEV_1_, following ivacaftor treatment was reported in studies evaluating within-group change. Increases were observed as early as 1 month after initiation of ivacaftor compared to baseline, with ppFEV_1_ generally remaining above baseline through all time points reported, regardless of study design and patient characteristics (Figure 3) [30,32,34,37,40,41,42,44,45,47,49,51,53,54,55,57,58,61,62,64,66,67,71,72,73,74,75,76,77,78,79,80,82,83,84,85,86,87,88,91,92,93,94,95,96,97,98,99,102]. Specifically, in the majority of studies reporting 12 months of follow-up, the mean absolute change in ppFEV_1_ from baseline with ivacaftor treatment increased by 5–10 percentage points in most studies (mean range: –1.4 to 15.0 percentage points) (Figure 3) [30,34,40,44,49,51,53,55,57,62,66,71,73,75,76,83,91,93,95,96,98,99].

Results from the LTSS disease progression cohorts showed better preserved lung function with ivacaftor treatment relative to a modulator untreated comparator cohort [30]. Better preserved lung function was reported for the ivacaftor-treated cohort than modulator untreated comparator cohorts from baseline for up to 5 years in the US cohort (change in ppFEV_1_ −0.7 (95% confidence interval (CI) −1.6, 0.2) vs. −8.3 (−9.0, −7.7) percentage points) and up to 4 years in the UK cohort (4.9 (3.30, 6.56) vs. −4.3 (−5.06, −3.44) percentage points), regardless of age or baseline disease severity [30].

Lung function following ivacaftor treatment in pwCF with severe lung disease (i.e., ppFEV_1_ <40%) was assessed in two studies with comparator groups [32,37]. The first, the Australian compassionate-use study that included ivacaftor-treated pwCF (*n* = 17) with severe lung disease and matched modulator untreated individuals (*n* = 314) showed an immediate significant improvement from baseline to the first 2–3 months with ivacaftor therapy versus modulator untreated individuals (absolute change, 11.9 (SD 8.6) vs. 0.5 (SD 9.2) percentage points; *p* < 0.001), which was sustained at 1 year (absolute change, 10.3 (SD 8.1) vs. −0.4 (SD 9.1) percentage points; *p* < 0.001) [37]. Similarly, results from the UK and Ireland compassionate-use study showed a significantly greater increase in median, within-patient, absolute change in ppFEV_1_ from baseline in the ivacaftor-treated group versus a modulator untreated comparator group over a median follow-up of 8 months (3.8 (interquartile range (IQR) 0.2–7.7) vs. 0.6 (IQR −2.1 to 2.8) percentage points; *p* = 0.009) [32].

#### 3.2.2. PEx

Results from seven studies evaluating within-group change showed consistent decreases in PEx following initiation of ivacaftor therapy compared with pre-ivacaftor initiation [30,40,44,49,53,66,96]. PEx decreased from the 12 months pre- to the 12 months post-initiation of ivacaftor in the following studies: BRIO (interim analysis data *n* = 107; 0.97 vs. 0.51 per patient per year (PPPY); rate ratio (RR) 0.53; 95% CI 0.40, 0.69) [53], CORK (*n* = 33; 0.88 vs. 0.21 PPPY; *p* = 0.006) [66], GOAL (*G551D* cohort *n* = 151; 0.57 PPPY decrease over follow-up; 95% CI −0.78, −0.36; *p* < 0.001) [44], VOCAL (interim analysis *n* = 71; non-*G551D* cohort; 0.81 vs. 0.24 PPPY; RR 0.29; 95% CI 0.15, 0.56) [40], and US CF Foundation Patient Registry (CFFPR) (annual decrease of 1.2 PEx) [49] studies.

Furthermore, in the cross-sectional analysis of the LTSS in 2014, there was a significantly lower risk of PEx in the ivacaftor-treated versus modulator untreated comparator cohorts in the US and UK cohorts. The trend for lower annual risk of PEx in the ivacaftor-treated versus modulator untreated comparator cohort in the US and UK cohorts was similar across age groups (0 to <6 (not significant in UK cohort), 6 to <12, 12 to <18, and ≥18 years) or baseline ppFEV_1_ (<40% (not significant in UK cohort), 40% to <70%, ≥70%) stratum [29].

A study (*n* = 13) that evaluated the rate of PEx in pwCF with severe lung disease (ppFEV_1_ <40% in the preceding 6 months, or being on a lung transplant waiting list and/or having a severely worsening trend of lung function (ppFEV_1_ loss >10% during the previous year)) and non-*G551D* gating mutations reported that PEx decreased from 4.38 PPPY in the period 12 months before ivacaftor initiation to 2.15 PPPY in the 12 months post-initiation of ivacaftor therapy [96].

### 3.3. Nutritional Parameters

**Figure 4:**
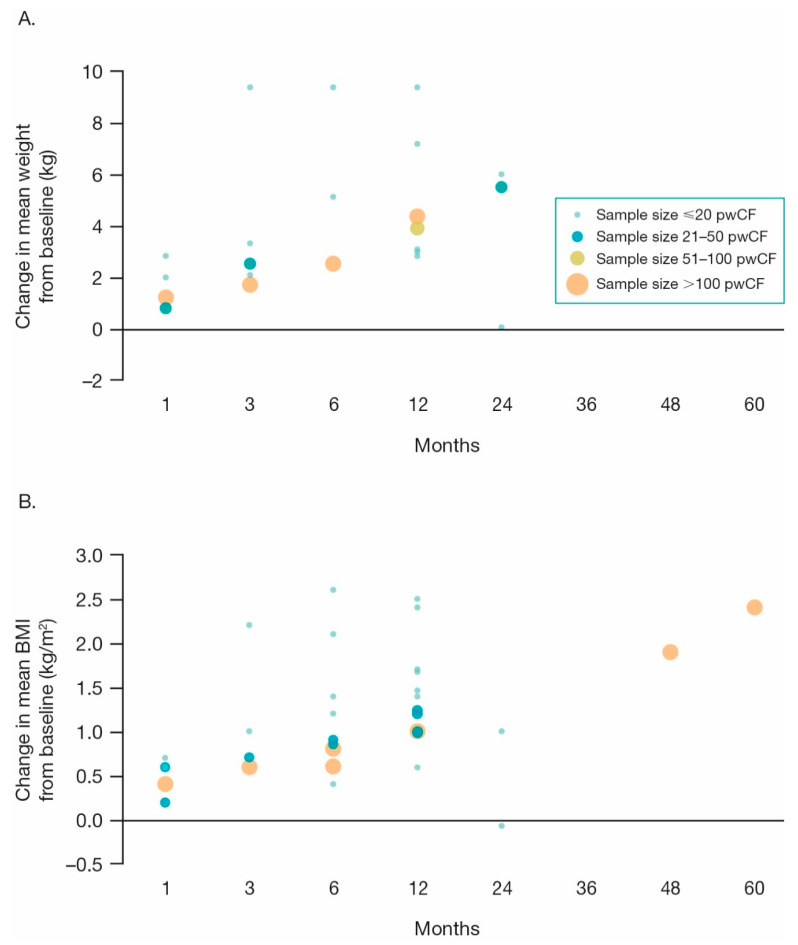
Change in mean weight (**A**); 10 studies) and mean BMI (**B**); 17 studies) with ivacaftor treatment [30,34,37,40,42,44,45,46,49,51,53,58,66,69,71,72,73,75,78,83,86,92,96,98,99,101]. Note: Figure only includes studies reporting absolute change from baseline within the ivacaftor-treated cohort in BMI and/or weight at 1, 3, 6, 12, 24, 36, 48, and/or 60 month time points following ivacaftor treatment. BMI: body mass index; pwCF: people with cystic fibrosis.

Across studies that evaluated within-group change, increases in weight [32,37,42,45,46,49,51,72,75,78,84,92,96,97,98,99,101,102] and BMI [30,32,34,37,40,41,42,44,45,46,47,53,58,64,66,69,71,72,73,75,79,83,86,92,94,95,96,98,99,101] were consistently noted following the initiation of ivacaftor therapy, starting as early as 1 month and improving over time as seen in studies with longer follow-up periods (Figure 4). Weight increased from baseline by 0.8–2.8 kg at 1 month [42,45,46,96] and by 2.8–7.2 kg at 12 months following initiation of ivacaftor [37,49,51,96,98,99]. BMI was observed to increase from baseline by 0.2–0.7 kg/m^2^ at 1 month [42,45,46,95,96] and by 0.6–2.4 kg/m^2^ at 12 months [34,37,39,44,53,66,71,73,75,83,95,96,98,99].

Changes in BMI *z*-score with ivacaftor treatment in pwCF aged <20 years were reported in two multinational (i.e., VOCAL interim analysis [40], *n* = 71; US, Canada, Italy [72], *n* = 23), one national (BRIO interim analysis [53], *n* = 107), and one single-center study enrolling pediatric patients (United Arab Emirates [57], *n* = 12), with follow-up ranging from 3–12 months. A significant mean (SD) increase of 0.3 (0.4) points in BMI *z*-score from baseline to 3 months with ivacaftor treatment was reported in one study (*p* < 0.01) [72]; a mean increase of 0.29–0.6 points from baseline to 12 months was reported in three studies, which was significant in two studies (*p* < 0.0001 each), and significance was not reported in one study [40,53,57].

A significant increase in BMI from baseline through 12 months for ivacaftor-treated pwCF aged 2–17 years compared to a modulator untreated comparator group was reported in pwCF attending the pediatric CF center at Cork University Hospital (0.98 ± 0.51 kg/m^2^ vs. comparator (BMI not reported); *p* = 0.010) [34]. In the LTSS disease progression cohort, the increase in BMI from baseline to Year 5 for the US cohort and from baseline to Year 4 in the UK cohort was greater in the ivacaftor-treated cohort versus modulator untreated comparator cohorts (US: overall, BMI 2.4 vs. 1.6 kg/m^2^; pediatric, BMI percentile 6.0 vs. −3.4; adult, BMI 1.3 vs. 0.7 kg/m^2^ and UK: overall, 1.9 vs. 0.9 kg/m^2^; pediatric, 10.0 vs. −1.0 kg/m^2^; adult, 1.2 vs. 0.2 kg/m^2^) [30]. In a study using data from the UK and Ireland compassionate-use program, a non-significant increase from baseline in BMI over a median follow-up of 8 months was observed for the ivacaftor-treated cohort compared to the modulator untreated comparator cohort (0.84 vs. 0.2 kg/m^2^; *p* = 0.234) [32]. Similar trends were noted for weight, with a significant increase from baseline in the ivacaftor-treated versus the modulator untreated comparator cohort at 2–3 months (mean (SD) increase, 2.5 (2.6) vs. 0.3 (2.0) kg; *p* < 0.018) and at 12 months (2.8 (5.0) vs. 0.8 (3.3) kg; *p* = 0.02) in the Australian study [37] and a non-significant increase in ivacaftor-treated versus modulator untreated comparator cohort from 3 months prior to ivacaftor initiation over a median follow-up of 8 months post-initiation in the UK–Ireland study (median change, 2.3 kg (range, −0.4 to 4.2) vs. 0.6 kg (−0.5 to 3.2); *p* = 0.25) [32].

In pwCF with severe lung disease receiving ivacaftor through the Australian compassionate-use program, there was a significant increase from baseline in BMI versus the modulator untreated comparator cohort at 2–3 months (0.9 vs. 0.1 kg/m^2^; *p* < 0.001), which was maintained at 12 months (0.9 vs. 0.2 kg/m^2^; *p* = 0.02) [37].

### 3.4. CFRD and Exocrine Pancreas

#### 3.4.1. Exocrine Pancreas

The impact of ivacaftor on the use of pancreatic enzyme replacement therapy (PERT) was evaluated in a single pediatric study from Cork University Hospital (*n* = 28) [34]. This study found a decrease in mean PERT consumption for the ivacaftor-treated compared to the modulator untreated comparator cohort after 12 months of ivacaftor treatment (*p* = 0.039) [34]. Additionally, a subgroup analysis of this study (*n* = 7) reported a significant relative improvement in fecal elastase-1, a biomarker of pancreatic exocrine function, at 12 months from baseline (*p* = 0.013) [34].

#### 3.4.2. CFRD

Results from a single-center UK study (*n* = 24) showed pwCF receiving ivacaftor had an absolute decrease in glycated hemoglobin (HbA1c) of 3.0 mmol/L (*p* = 0.004) from baseline at 6 months [59]. In a subset of pwCF with normal glucose tolerance (*n* = 16), significant absolute decreases in HbA1c compared to baseline were observed at multiple time points: 2.1 mmol/L at 3 months (*p* = 0.027), 2.4 mmol/L at 6 months (*p* = 0.002), and 1.9 mmol/L at 12 months (*p* = 0.03) [59]. In contrast, a single-center study from Ireland in adults with CF (*n* = 24) on ivacaftor therapy found no significant change in fasting glucose, 2-h post-prandial glucose, or HbA1c from baseline to an unspecified time point [65].

The cross-sectional analysis of the LTSS showed that the prevalence of CFRD in 2014 was significantly lower for the ivacaftor-treated cohort than the modulator untreated comparator cohort in the US cohort (30.4% vs. 39.5%; *p* < 0.001; RR 0.77; 95% CI 0.70, 0.84) and the UK cohort (20.7% vs. 29.1%; *p* < 0.007; RR 0.71; 95% CI 0.58, 0.87) [29]. Analysis of the disease progression cohorts of the LTSS showed that the prevalence of CFRD for both the ivacaftor-treated and modulator untreated comparator cohorts increased over 5 years from baseline in the US cohort (increases of 12.1 and 18.3 percentage points) and over 4 years from baseline in the UK cohort (increases of 2.4 and 8.2 percentage points) [30]. However, the increase in prevalence of CFRD was lower in the ivacaftor-treated than in the modulator untreated comparator cohort in the US cohort at 5 years and in the UK cohort at 4 years [30].

### 3.5. Healthcare Utilization

#### 3.5.1. All-Cause Hospitalizations

Across studies that evaluated within-group change, all-cause hospitalizations reported using a variety of measures (e.g., patients (number, %) hospitalized, hospitalizations PPPY) decreased following initiation of ivacaftor therapy, with study follow-up ranging from 6–60 months [30,37,38,39,42,44,45,48,50,51,52,54,56,73,75,99].

In the GOAL study, mean (SD) hospitalizations PPPY decreased from 0.7 (1.2) in the 6 months before ivacaftor to 0.30 (1.2) in the 6 months after ivacaftor initiation, with a paired rate reduction of 0.35 hospitalizations PPPY (95% CI 0.19, 0.52; *p* < 0.001) [45]. The study also reported a significant decrease in the rate of hospitalization from the 12 months before ivacaftor initiation to 12 months after ivacaftor treatment initiation (−0.45 hospitalizations PPPY; 95% CI −0.60, −0.30; *p* < 0.001) [44]. Interim analyses of the VOCAL study showed a 62% reduction in the rate of hospitalization (0.44–0.17 PPPY; RR 0.38; 95% CI 0.15, 0.94) [39] and interim analysis of the BRIO study showed a 60% reduction (0.60–0.24 PPPY; RR 0.40; 95% CI 0.26, 0.61) [52], from 12 months before to 12 months after ivacaftor initiation. Results from four US administrative claims analyses showed reductions in the number of pwCF with hospitalizations: one study (*n* = 102) reported a 54% reduction from 6 months pre- to 6 months post-ivacaftor initiation (23.5% vs. 10.8%; *p* = 0.012) [38] and the remaining three studies reported reductions of 50% (*n* = 79; 32.9% vs. 16.5%; *p* = 0.021) [56], 55% (*n* = 143; 31% vs. 14%; *p* < 0.01) [48], and 56% (*n* = 84; 29.8% vs. 13.1%; *p* = 0.006) [50] from 12 months pre- to 12 months post-ivacaftor initiation. One study reported similar results regardless of age, with the number of pwCF hospitalized among those aged 6–17 years decreased by 61% (*n* = 53; 34% vs. 13%; *p* < 0.01) and the number of pwCF hospitalized among those aged 18–64 years decreased by 50% (*n* = 90; 29% vs. 14%; *p* < 0.001) from 12 months pre- to 12 months post-ivacaftor initiation [48]. Significant decreases in the number of pwCF with CF-related hospitalizations were also reported in two studies: one study reported a 67% decrease (*n* = 79; 19.0% vs. 6.3%; *p* = 0.041) [56] and the other study reported a 78% decrease (*n* = 143; 16% vs. 4%; *p* < 0.01) [48], from 12 months pre- to 12 months post-ivacaftor initiation.

Results from the LTSS for the US disease progression cohort showed a significantly lower risk of all-cause hospitalizations for the ivacaftor-treated than the modulator untreated comparator cohort beginning at Year 1 (25.5% vs. 37.4%; RR 0.68; 95% CI 0.59, 0.79) and continuing through the end of follow-up at Year 5 (26.3% vs. 44.3%; RR 0.59; 95% CI 0.52, 0.68) [30].

For pwCF with severe lung disease, the Australian registry study showed an 85% decrease in the median (IQR) number of hospital admissions for those treated with ivacaftor at 12 months following treatment compared to the 12-month period prior to treatment (0.6 (0.0–1.8) vs. 4.0 (2.0–6.0) admissions; *p* < 0.001). This study also showed a 75% lower median number of hospital admissions for pwCF receiving ivacaftor versus modulator untreated comparator cohort at 12-month follow-up from 12 months prior to treatment (0.6 (0.0–1.8) vs. 2.4 (0.6–3.5) admissions; *p* = 0.007) [37].

#### 3.5.2. PEx-Related Hospitalizations

Three studies that evaluated within-group change reported decreases in PEx-related hospitalizations following ivacaftor therapy [30,39,50,53]. The interim analysis of the VOCAL study showed a 79% reduction (0.33–0.07 PPPY; RR 0.21; 95% CI 0.08, 0.54) [39] and the interim analysis of the BRIO study showed a 70% reduction (0.22–0.07 PPPY; RR 0.30; 95% CI 0.15, 0.57) in the rate of PEx-related hospitalizations, from 12 months pre- to 12 months post-ivacaftor initiation [53]. A US administrative database study (*n* = 84) also reported a significant 48% decrease in the proportion of pwCF hospitalized for PEx following ivacaftor initiation, from 12 months pre- to 12 months post-ivacaftor initiation (25.0% vs. 13.1%; *p* = 0.033) [50].

In the UK disease progression cohort of the LTSS, the ivacaftor-treated cohort reported a lower risk of PEx-related hospitalizations compared to a modulator untreated cohort at Year 1 (36.0% vs. 41.7%; RR 0.86; 95% CI 0.72, 1.03), and at the end of follow-up in Year 4 (26.3% vs. 44.6%; RR 0.59; 95% CI 0.47, 0.73) [30].

#### 3.5.3. Antibiotic Utilization and Duration

Decreases in the usage and duration of antibiotics following initiation of ivacaftor therapy were reported in multiple studies evaluating within-group change [32,50,51,52,54,67,71,76,83,90,91,94,95,97,99,100,102].

Reductions in acute antibiotic use for PEx were reported in the interim analyses of the VOCAL and BRIO studies from 12 months pre- to 12 months post-ivacaftor initiation; the VOCAL study showed a 77% reduction in acute antibiotic use for PEx (1.28 vs. 0.29 courses PPPY; RR 0.23; 95% CI 0.12, 0.44) [39] and the BRIO study showed a 53% reduction (1.59 vs. 0.75 courses PPPY; RR 0.47; 95% CI 0.32, 0.68) [52]. Results from the Ireland CF registry analysis showed a 46% decrease in the number of IV antibiotics (0.61 vs. 0.33 courses PPPY; *p* < 0.01) and a 49% decrease in the number of oral antibiotics (2.14 vs. 1.10 courses PPPY; *p* < 0.01) from 12 months pre- to 12 months post-ivacaftor therapy [54]. Results from an analysis of US claims data found a significant decrease in the number of pwCF on outpatient IV (44.0% vs. 22.6%; *p* = 0.012) and inhaled (47.6% vs. 36.9%; *p* = 0.039) antibiotics from the 12 months prior to the 12 months following ivacaftor initiation [50]. A significant 85% decrease in IV antibiotic use from 12 months pre- to 12 months post-ivacaftor therapy was reported in pediatric patients treated at four centers in the US (*n* = 26; *p* = 0.03) [67] and a 41% decrease from baseline to 48 weeks post-ivacaftor therapy at a single center in France (3.7 vs. 2.1 courses PPPY) [102]. Analysis of the French CF Registry data (*n* = 57) showed significant decreases in antibiotic use from the 12 months pre- to 24 months post-ivacaftor therapy for IV (0. 9 vs. 0.3 courses PPPY; *p* = 0.011), but not oral antibiotics (1.8 vs. 1.1 courses PPPY; *p* = 0.072) [51]. Non-significant decreases in oral, inhaled, and IV antibiotic courses, including use specifically for PEx, following ivacaftor treatment were reported in several smaller studies from the US and UK enrolling seven to 10 patients [90,97,99]. No significant difference in inhaled antibiotic use was found at baseline through 60 months of follow-up between patients treated with ivacaftor and modulator untreated comparators in an analysis of UK CF registry data (*p* values not reported) [31].

The interim analysis of the VOCAL study showed a 77% reduction in the duration of use of acute antibiotics for PEx (15.2 vs. 3.4 days PPPY; RR 0.23; 95% CI 0.11, 0.48), and a 46% reduction in the duration of acute antibiotics for PEx in the interim analysis of the BRIO study (20.9 vs. 11.4 days PPPY; RR 0.54; 95% CI 0.40, 0.72), from 12 months pre- to 12 months post-ivacaftor initiation [39,52]. Analysis of the French CF Registry data showed significant decreases in days of antibiotic use from the 12 months pre- to 24 months post-ivacaftor therapy for IV (12.7 vs. 5.4 days PPPY; *p* = 0.016), but not oral (37.2 vs. 23.6 days PPPY; *p* = 0.055) antibiotics [51]. Analysis of the Ireland CF registry showed a 44% decrease in IV antibiotic days (9.4 vs. 5.2; *p* < 0.01) and a 53% decrease in oral antibiotic days (33.6 vs. 15.9; *p* < 0.01) from 12 months pre- to 12 months post-ivacaftor therapy [54]. IV antibiotic days also significantly decreased by 88% in a UK single-center study (*n* = 11; 37.0 vs. 4.5 days per year; *p* < 0.001) [83] and by 70% in a UK study enrolling patients with an *R117H* mutation (*n* = 9; 43 vs. 13 days) from 24 months pre- to 24 months post-ivacaftor initiation [100].

Reductions in duration of IV antibiotic use for pwCF with severe lung disease were also reported. There was a 49% reduction following ivacaftor treatment compared to the 12 months prior to ivacaftor initiation (*n* = 21; 74 vs. 38 days per year; *p* = 0.0016) [32], a 57% reduction at 12 months post-ivacaftor compared to baseline (*n* = 12; 28 vs. 12 days per year; *p* = 0.042) [91], and an 80% reduction at 12 months after starting ivacaftor treatment compared to the 12 months before ivacaftor (*n* = 25; *p* value not reported) [71]. Results from a study in pwCF with severe lung disease also showed a significant difference in the median within-patient change from baseline over a median follow-up of 8 months of therapy between the ivacaftor and modulator untreated comparator cohort for both inpatient IV and total IV days (–14 vs. 1 day, *p* = 0.0006; –36 vs. 10 days, *p* = 0.003, respectively) [32].

### 3.6. Lung Microbiome

#### 3.6.1. *Pseudomonas aeruginosa*

The favorable impact of ivacaftor on *P. aeruginosa* prevalence was reported in studies evaluating within-group change [29,30,31,42,44,45,51].

The GOAL study showed decreased odds of a positive *P. aeruginosa* culture in pwCF with a *G551D* gating mutation (odds ratio (OR) 0.65; 95% CI 0.53, 0.79; *p* < 0.001) [44] and reduced risk of *P. aeruginosa* in pwCF with non-*G551D* gating mutations of 41.6% (95% CI not reported) [42] at 12 months of ivacaftor treatment compared to 12 months prior to ivacaftor initiation. A French CF registry study showed a significant decrease in positive *P. aeruginosa* cultures from baseline to 12 months (58.2% vs. 33.3%; *p* = 0.0005), while results from a UK registry study showed a slight increase in *P. aeruginosa* prevalence from 12 months before ivacaftor initiation to 12 months after ivacaftor initiation (46.4% vs. 47.9%) followed by a steady decrease to 35.9% through the end of follow-up at Year 4 [31,51]. Results from smaller studies from Israel, Oman, Ireland, and the UK that enrolled <25 pwCF showed little change in *P. aeruginosa* prevalence from the period before ivacaftor initiation to after ivacaftor initiation [70,73,76,90].

In the disease progression cohorts of the LTSS, the ivacaftor-treated cohort showed lower prevalence of *P. aeruginosa* compared to baseline and to the modulator untreated comparator cohort up to 5 years in the US cohort (ivacaftor: 56.5% baseline vs. 45.1% at Year 5; comparator: 50% baseline vs. 55.7% at Year 5) and up to 4 years in the UK cohort (ivacaftor: 63.2% baseline vs. 38.9% at Year 4; comparator: 57.2% baseline vs. 55.9% at Year 4) [30]. In a single-center study from the UK, pwCF treated with ivacaftor had a significantly lower rate of *P. aeruginosa* prevalence versus modulator untreated comparators at 12 months (adjusted prevalence ratio (PR) 0.78; 95% CI 0.68, 0.89; *p* < 0.001) and at 3 years post-ivacaftor initiation (adjusted PR 0.68; 95% CI 0.58, 0.79; *p* < 0.001) [31].

#### 3.6.2. *Staphylococcus aureus*

There was little to no reduction in *S. aureus* prevalence following ivacaftor initiation as reported in studies that evaluated within-group change [29,31,44,67,70]. The GOAL study in pwCF with a *G551D* gating mutation reported no significant difference in the odds of a positive methicillin-sensitive *S. aureus* (MSSA) (*p* = 0.59) or methicillin-resistant *S. aureus* (MRSA) (*p* = 0.44) culture from 24 months pre- to 12 months post-ivacaftor initiation [44]. Results from one study of pediatric pwCF (*n* = 26) noted that MRSA persisted in 80% of pwCF after 12 months of ivacaftor therapy [67], while another study showed no change in MSSA (OR 1.04; 95% CI 0.59, 1.84; *p* = 0.46) and a significant increase in the odds of positive MRSA cultures (OR 3.39; 95% CI 1.54, 7.48; *p* = 0.0025) from 24 months before ivacaftor initiation to 36 months after ivacaftor initiation [70].

The cross-sectional analysis of the LTSS showed a trend toward lower prevalence of *S. aureus* in the ivacaftor-treated versus the modulator untreated comparator cohort in 2014 (63.9% vs. 69.9%; *p* < 0.0001 in the US cohort and 29.8% vs. 33.9%; *p* = 0.1706 in the UK cohort) and of MRSA (23.3% vs. 29.4%; *p* < 0.0001 in the US cohort and 2.7% vs. 3.7%; *p* = 0.42 in the UK cohort) [29]. Results from the UK CF registry analysis also showed a lower prevalence of *S. aureus*-positive cultures in the ivacaftor-treated cohort versus modulator untreated comparator cohort beginning in Year 1 following ivacaftor initiation (30.1% ivacaftor-treated vs. 32.1% comparator; adjusted PR 0.92; 95% CI 0.76, 1.1; *p* = 0.39) and persisting through Year 3 (30.1% vs. 34.8%; adjusted PR 0.85; 95% CI 0.7, 1.01; *p* = 0.08); however, only the difference at Year 2 was significant (25.7% vs. 32.9%; adjusted PR 0.77; 95% CI 0.62, 0.94; *p* = 0.01) [31].

#### 3.6.3. Other

In studies evaluating within-group change following initiation of ivacaftor, there was no significant change in the prevalence of *B. cepacia* complex or *Stenotrophomonas maltophilia* compared with baseline [31,44,70]. The cross-sectional analysis of the LTSS showed a trend toward a lower prevalence of pulmonary microorganisms in the ivacaftor-treated compared to modulator untreated comparators in the US and UK cohorts in 2014; for instance, the prevalence of *S. maltophilia* was lower in the ivacaftor-treated compared to modulator untreated comparator cohort in the US cohort (10.8% vs. 15.0%; *p* < 0.0002) and in the UK cohort (5.1% vs. 8.2%; *p* = 0.0522) [29].

A significant decrease in prevalence of *Aspergillus* spp. was reported in the GOAL study in pwCF with a *G551D* gating mutation after 12 months of ivacaftor treatment compared to 12 months before initiation (*p* = 0.04) [44].

The cross-sectional analysis of the LTSS showed a lower prevalence of *Aspergillus* spp. in both the US and UK ivacaftor-treated cohorts compared to modulator untreated comparator cohorts in 2014 (US: 10.7% vs. 18.8%; *p* < 0.0001; UK: 10.3% vs. 20.2%; *p* < 0.001) [29]. Similarly, results from the UK Registry analysis showed significantly lower prevalence of *Aspergillus* spp. in an ivacaftor-treated cohort than modulator untreated comparators at Year 1 (11.6% vs. 19.9%; adjusted PR 0.56; 95% CI 0.4, 0.77; *p* < 0.001), Year 2 (11.2% vs. 19.7%; adjusted PR 0.56; 95% CI 0.39, 0.77; *p* < 0.001), and Year 3 (4.7% vs. 16.9%; adjusted PR 0.27; 95% CI 0.15, 0.44; *p* < 0.001) of follow-up [31].

### 3.7. PROs

#### 3.7.1. CFQ and CFQ-R Respiratory Symptom Scores

The CFQ-R is a validated, disease-specific PRO instrument that measures nine QoL domains and three symptom scales in pwCF, where higher scores indicate better health [105].

Sustained improvements were consistently reported for the CFQ-R respiratory domain in studies evaluating within-group change following ivacaftor treatment as early as 1 month after initiation of ivacaftor (compared to baseline) and continuing through reported time points in studies with longer follow-up periods [41,42,45,46,66,83]. The GOAL study in pwCF with a *G551D* mutation, including the extension study, showed significant improvement in the CFQ-R respiratory domain score from baseline at 1 month (mean increase = 9.7 points; *p* < 0.001), at 6 months (mean increase = 7.4 points; *p* < 0.001) [45], and at 66 months (mean increase = 6.7 points; *p* = 0.002) of ivacaftor treatment [41,45]. Similar results in the CFQ-R respiratory domain scores were seen in pwCF with non-*G551D* gating mutations (14.2-point mean increase from baseline at 6 months; *p* = 0.0366) [42] and in pwCF with the *R117H* mutation participating in the GOAL-e2 study (9-point mean increase from baseline at 1 month; *p* = 0.02) [46]. Significant increases in the mean values for CFQ-R respiratory domain scores from baseline at 12 months were reported in two studies; a single-center study from Wales (*n* = 11; 26.3-point mean increase; *p* = 0.004) [83] and among adult pwCF in the CORK study (*n* = 20; 17.5-point mean increase; *p* < 0.001) [66]. PwCF aged 6–14 years in the CORK study (*n* = 13) noted a mean increase of 8.8 points (*p* = 0.08) from baseline at 12 months following ivacaftor treatment in the caregiver-completed CFQ-R respiratory domain score [66].

#### 3.7.2. Other PROs

The SNOT-20 is a validated PRO that measures overall quality of health using 20 questions on nose, sinus, and general health-related items; scores range from 0 (no problem on any of the measured symptoms) to 100 (worst score for all measured symptoms) [106]. The GOAL study showed low SNOT-20 scores at baseline for pwCF with *G551D* and pwCF with non-*G551D* gating or *R117H* mutations (0.9 and 1.1, respectively), reflecting very mild to no problems at baseline. Even though baseline scores in this population were already low, following ivacaftor treatment scores decreased by 0.20 points at 6 months in pwCF with *G551D* mutations and by 0.30 points at 1 month in pwCF with non-*G551D* gating or *R117H* mutations [42,45]. Two studies from a single center in Italy (*n* = 18 and *n* = 13) reported significant improvements in the 6-min walk test distance following 6 months of ivacaftor therapy with mean (SD) distances increasing from 535.1 (87.1) m before ivacaftor treatment to 611.6 (66.0) m after 12 months of ivacaftor treatment (*p* = 0.002) [96]. Results from the CORK study (*n* = 33) showed improvement in the modified shuttle walk test (i.e., an increase of 109 m) from baseline up to 12 months of ivacaftor treatment [66].

### 3.8. Adverse Events

Among the seven studies that reported adverse events with ivacaftor treatment, with follow-up ranging from 8–24 months, no new safety concerns were identified [32,40,53,71,95,96,100]. Adverse events were generally consistent with the known safety profile of ivacaftor and clinical manifestations of CF relative to the age of the patients included. Two studies assessed the relationship between adverse events and ivacaftor treatment: the proportion of adverse events considered to be related to ivacaftor was 0% at a median follow-up of 8 months in one study [32] and the other study reported most adverse events (62/73) not related to ivacaftor [53].

### 3.9. Other CF-Related Complications

Other CF-related complications were reported in the cross-sectional analysis of the LTSS [29]. In the US cohort in 2014, relative to the modulator untreated comparator cohort, the ivacaftor-treated pwCF had significantly lower prevalence of bone/joint (17.7% vs. 22.4%; RR 0.79; 95% CI 0.69, 0.90), depression (14.2% vs. 17.1%; RR 0.83; 95% CI 0.71, 0.96), and hepatobiliary (4.6% vs. 7.8%; RR 0.59; 95% CI 0.45, 0.77) complications. Similarly, in the UK cohort in 2014, ivacaftor-treated pwCF had significantly lower prevalence of bone/joint (18.2% vs. 27.7%; RR 0.66; 95% CI 0.53, 0.82) and hepatobiliary (22.4% vs. 28.0%; RR 0.80; 95% CI 0.66, 0.97) complications relative to the modulator untreated comparator cohort [29].

### 3.10. Organ Transplants

The interim analysis of the BRIO study reported one lung transplantation during the 12-month period following ivacaftor initiation and the interim analysis of the VOCAL study reported no transplantations [40,53].

PwCF in the US disease progression cohort of the LTSS receiving ivacaftor had a higher probability of remaining transplant free over 5 years compared with the modulator untreated comparator cohort (lung transplants: 11/805 vs. 133/3815; *p* value not reported), and pwCF in the UK disease progression cohort treated with ivacaftor had a non-significant higher probability of remaining transplant free over the course of 4 years compared with the modulator untreated comparator cohort (lung transplants: 1/293 vs. 28/1433, *p* value not reported) [30].

Additionally, results from the UK and Ireland compassionate-use study found a lower occurrence of organ transplant for pwCF with severe lung disease receiving ivacaftor versus the modulator untreated comparator cohort over a median follow-up of up to 8 months (0% vs. 5.7%) [32] and 38 months (4.8% vs. 22.9%) [33], and ivacaftor was associated with a lower rate of transplantation compared with the modulator untreated comparator cohort at a median follow-up of 38 months (hazard ratio 0.127; 95% CI 0.038, 0.429; *p* = 0.001) [33].

### 3.11. Mortality

During the 12-month period following ivacaftor initiation, the interim analysis of the BRIO study reported no deaths and the interim analysis of the VOCAL study reported one death (unrelated to ivacaftor treatment) [40,53].

PwCF receiving ivacaftor versus the modulator untreated comparator cohort in the disease progression cohort of the LTSS had a higher probability of survival over 5 years (deaths: 17/805 vs. 160/3815; *p* value not provided) in the US cohort and a numerically higher probability of survival over 4 years (deaths: 8/293 vs. 54/1433; *p* value not provided) in the UK cohort [30].

A study enrolling adult pwCF with severe lung disease and a *G551D* mutation (*n* = 14) reported three deaths during an 8-month follow-up post-ivacaftor initiation [84] and another study enrolling pwCF with severe lung function and non-*G551D* gating mutations (*n* = 13) reported no deaths during the 12-month period post-ivacaftor initiation [96]. Results from the UK and Ireland compassionate-use program that enrolled pwCF with severe lung disease showed no deaths in either the ivacaftor-treated or modulator untreated comparator cohort at a median follow-up of 8 months [32]. However, by a median follow-up of 38 months, treatment with ivacaftor was associated with a significantly lower rate of death compared with the modulator untreated comparator cohort (hazard ratio 0.24; 95% CI 0.059, 0.984; *p* = 0.047) [33].

## 4. Discussion

Our review identified 57 observational studies providing real-world evidence on the effectiveness and safety of ivacaftor. These real-world studies were conducted in a variety of geographic settings and ranged in size from large multinational and registry studies that enrolled dozens or even hundreds of pwCF to smaller single-center studies that enrolled as few as six pwCF. The pwCF populations studied had differences in disease severity and indicated ivacaftor-responsive mutations, such as *G551D*, non-*G551D* gating, and *R117H* mutations.

The current study is broadly consistent with previous reviews of clinical trial data for ivacaftor. A previous systematic review by Whiting et al. (2014) examined the clinical efficacy and safety of CFTR modulators, including ivacaftor, in randomized controlled trials [107]. The authors documented improvements in pulmonary function (ppFEV_1_), decreases in PEx rates, increases in weight and BMI, and improvements in QoL in pwCF with a *G551D* mutation treated with ivacaftor. In addition, a recent review of phase 2 and phase 3 clinical trials of CFTR modulators through 2020 reported that pwCF with the most beneficial effects from CFTR modulation in terms of lung function, PEx, and symptom improvement were those with one *CFTR* gating mutation who received ivacaftor [108]. A review summarizing published literature from clinical trials and case series reported a beneficial impact of CFTR modulators on clinical outcomes for pwCF with severe lung disease [109]. Similar to these systematic reviews of clinical trial data, our review of real-world observational studies found improvements in pulmonary function, decreases in PEx and hospitalizations, improvements in nutritional parameters and CF-related complications, and improvements in QoL in pwCF receiving ivacaftor. Although improvements in outcomes varied in magnitude across studies, trends were generally consistent despite diverse geographies, target populations, study designs, and follow-up periods. Qualitative evidence from this review also demonstrates that across real-world studies, ivacaftor treatment led to clinical benefits that were consistent with those observed in the ivacaftor clinical trial program [16,20,24] and were sustained over time based on studies with extended follow-up duration (up to 5 years). Furthermore, the qualitative evidence from this review in pwCF with severe lung function treated with ivacaftor that showed clinical benefit on pulmonary function, PEx, weight, and antibiotic duration corroborates the published evidence. Although safety reporting of ivacaftor in real-world studies was not the primary objective of this study, we found that safety results from real-world studies were consistent with the established safety profile of ivacaftor from clinical trials (such as cough, headache, nasal congestion, upper respiratory tract infection, diarrhea), providing evidence that ivacaftor is generally safe and well tolerated during long-term treatment. This finding is consistent with a recently published systematic literature review summarizing adverse events observed in real-world studies for CFTR modulators, which reported that the majority of adverse events in real-world studies for ivacaftor were consistent with those observed in clinical trials [110].

### 4.1. Clinical Implications

The predicted life expectancy for pwCF undergoing symptomatic treatment management is decades shorter than that of the general population [13,14,111,112,113]. PwCF experience a decline in lung function over time and respiratory failure is the primary cause of death [3,4]. Results from the identified real-world studies consistently demonstrated improvement in lung function with ivacaftor therapy. Improvement in lung function following ivacaftor initiation was noted within 1 month and maintained for up to 5 years of follow-up. While the LTSS that followed pwCF for 5 years showed a slight decline in ppFEV_1_ in the ivacaftor-treated cohort over the course of the follow-up period, this decline was likely attributable to the progressive nature of CF disease; this interpretation is strongly supported by the comparatively better-preserved lung function in the ivacaftor-treated cohort than modulator untreated comparator cohort.

Natural history data from CF registries in the US, Europe, Australia, and Canada show low BMI, high number of PEx and hospitalizations, and high prevalence of CF-related comorbidities among pwCF treated with symptomatic care [4,13,14,111,112,114]. In contrast, our review of real-world studies of pwCF treated with ivacaftor found consistent increases in weight and BMI, and decreases in PEx. Treatment with ivacaftor also showed favorable trends in reducing the prevalence of *P. aeruginosa* and the prevalence of CF-related complications, including CFRD, bone/joint complications, hepatobiliary complications, depression, and improvement in CFQ-R and other PROs, although few studies evaluated these outcomes. Results from studies with modulator untreated comparators provide further evidence of improvement in nutritional parameters, CF-related complications, and *P. aeruginosa* prevalence, and PROs associated with ivacaftor treatment [28,29,30,31,32,34,35,37,58]. Reductions in healthcare resource utilization such as hospitalizations, antibiotic use, and antibiotic duration with ivacaftor were observed in real-world studies from different healthcare systems, both relative to a modulator untreated comparator and in studies that compared periods before and after initiation of ivacaftor in a cohort of pwCF. Reductions in PEx and healthcare resource use are important indicators of burden of disease for pwCF, caregivers, and healthcare systems.

Based on a study predicting mortality in pwCF by Liou et al. [11], as described earlier, four of the nine patient characteristics reported in the Cox proportional hazard model are impacted on by ivacaftor treatment. While results from the clinical program provide evidence for the impact of ivacaftor on three of nine patient characteristics (ppFEV_1_, weight-for-age, and PEx), results from this systematic literature review support improvements in those characteristics and additionally provide information on the positive impact of ivacaftor on diabetes. Evidence from the clinical trial program in combination with the real-world systematic review evaluated in context of the Liou et al. model suggests ivacaftor may have a beneficial effect on survival [11]. Empirical evidence from real-world studies have shown a positive impact on survival, a reduction in organ transplantation, and improved transplant-free survival in pwCF treated with ivacaftor.

In summary, the improvement in outcomes associated with real-world ivacaftor use summarized in this study demonstrate the substantial benefit of CFTR modulation with ivacaftor on pulmonary and non-pulmonary outcomes, many of which were not studied in clinical trials. The real-world evidence collectively supports the value of ivacaftor as a long-term treatment that can fundamentally modify the progression of CF by treating the underlying cause of the disease.

### 4.2. Limitations

As the goal of this review was to include as wide a range of studies as possible, exclusion criteria were limited. Differences in study designs and reported outcomes make formal comparisons or synthesis of data across studies infeasible. In addition, the included studies did not evaluate all outcomes uniformly and outcome definitions varied across studies. Furthermore, prescription patterns for treating CF symptoms such as PEx with antibiotics vary by physician and geographic setting, which could explain the variation observed in the reduction of antibiotic usage and duration following ivacaftor treatment across studies. Publications also did not provide statistical tests for all comparisons, either between groups or across time periods. For certain outcomes, results were reported as absolute change from baseline at follow-up time periods within the ivacaftor-treated cohort, which is different from how results are reported in the pivotal clinical trials for ivacaftor, usually calculated as mean of differences between ivacaftor-treated and best supportive care arms. Moreover, selection of results for publication from each study may have been subject to bias that cannot be evaluated. We also note that the ongoing VOCAL and BRIO studies reported interim results and, therefore, are subject to change. No studies reported data on productivity or work/school days lost to quantify the societal impact of ivacaftor. We further note that a number of real-world studies examined pwCF from the same large CF centers and national registries, and, therefore, some pwCF may have contributed data to multiple studies. In addition, only a few studies reported in this review included a modulator untreated comparator cohort, making it difficult to fully evaluate the role of natural disease progression on the observed treatment impact. Notably, studies with a modulator untreated comparator group provided evidence of improved disease course for ivacaftor-treated versus modulator untreated patients. However, because most studies reported outcomes before and after ivacaftor initiation, and CF is characterized by disease progression over time, the true treatment effect of ivacaftor is likely to be underestimated. Despite these limitations, the improvement in outcomes noted in this literature review of real-world evidence is consistent with results observed in ivacaftor clinical trials.

### 4.3. Future Research Directions

There remains a need to understand the multi-systemic long-term benefits of ivacaftor treatment, as well as broader humanistic and societal benefits; therefore, additional long-term studies evaluating ivacaftor use in pwCF are warranted. Given the progressive damage to the lungs, digestive system, and other organs due to CF, disease progression among adults treated with ivacaftor may not be completely reversed; however, treatment with ivacaftor has been shown to reduce the rate of annual lung function decline [115]. Preliminary evidence suggests early initiation of ivacaftor may reverse existing pancreatic damage and prevent or delay further damage to pancreatic and airway functioning [21]. Hence, real-world research on the impact of ivacaftor in pediatric populations is of great interest. Importantly, there is similarity in the efficacy of clinical endpoints observed between the ivacaftor and the recently approved elexacaftor/tezacaftor/ivacaftor clinical programs. In fact, elexacaftor/tezacaftor/ivacaftor demonstrated better efficacy in clinical endpoints among eligible pwCF in clinical trials with larger sample sizes than ivacaftor [116,117]. The consistency and durability of impact observed following ivacaftor treatment in this study provides some indication about the potential transferability of this impact for pwCF treated with elexacaftor/tezacaftor/ivacaftor. Future studies evaluating the impact of elexacaftor/tezacaftor/ivacaftor in real-world settings will be key in providing additional evidence on clinical benefits observed with highly effective modulator treatments.

## 5. Conclusions

This systematic literature review of real-world studies encompassing a range of study designs and data sources, geographic settings, patient characteristics, and study durations consistently identified improvements in a broad range of outcomes for pwCF receiving ivacaftor. Substantial treatment benefits were observed in lung function, nutrition, healthcare utilization, *P. aeruginosa* prevalence, and QoL, as well as in reduced mortality and organ transplantation. Improvements were evident as soon as 1 month after ivacaftor initiation and sustained for up to 5 years of follow-up. Overall, no new safety concerns were identified from these real-world studies. Our findings support long-term ivacaftor use to improve clinical markers of disease severity, slow the progression of CF, and potentially reduce disease burden, while allowing pwCF to have better survival outcomes and improved QoL.

## Figures and Tables

**Table 1:** Study and patient characteristics.

Reference(s) (Study Name)	Country/ Geographic Region	Data Type	Source	Follow-Up, Months	pwCF Treated With Ivacaftor, *n*	CFTR Mutation/Class of Mutation	pwCF Aged ≥18 Years, %	Mean (SD) Baseline ppFEV_1_, %
**Studies with non-ivacaftor-treated comparator**								
**>50** **pwCF treated with ivacaftor (3 studies)**								
Bell 2019 [28]	Interna-tional	Multicenter	NR (international)	22 (mean)	IVA: 72	*G551D*	58.3	79.8 (25.6)
					COMP: 137	*F508del*	69.3	70.7 (28.8)
Bessonova 2018 (LTSS) [29]	UK, US	Registry	US CF Foundation Patient Registry (CFFPR), UK CF Registry	UK, ≤24 ^a^ US, ≤36 ^a^	IVA: 411 (UK), 1256 (US)	Class I-VI and VI ^b^	62.3 (UK), 58.2 (US)	UK, 70.6 US, 79.8
					COMP: 2069 (UK), 6200 (US)		61.8 (UK), 58.2 (US)	UK, 71.4 US, 80.4
Volkova 2020 (LTSS) [30]				UK, ≤48 US, ≤60	IVA: 247 (UK), 635 (US)	Class I-III ^b^	69.2 (UK), 61.1 (US)	UK, 73.0 (23.6) US, 79.0 (25.3)
					COMP: 1230 (UK), 1874 (US)		66.6 (UK), 57.3 (US)	UK, 73.4 (22.4) US, 81.7 (23.7)
Frost 2019 [31]	UK	Registry	UK CF Registry	36	IVA: 276	*G551D*	NR	81.07 (22.5)
					COMP: 5296	Rest of CF population not treated with IVA		72.91 (23.3)
**20–50** **pwCF treated with ivacaftor (2 studies)**								
Barry 2014 [32], Barry 2015 ^c^ [33]	UK, Ireland	Multicenter	CF centers in UK and Ireland (Compassionate-use program)	37 (median)	IVA:21	*G551D*	100	26.5 (7.2) [32]
					COMP: 35	Non-*G551D* gating		30.3 (7.5) [32]
Emery 2019 ^c^ [34]	Ireland	Single center	Cork University Hospital	12	28 ^d^	NR	0	NR
**<20** **pwCF treated with ivacaftor (2 studies)**								
McLearn-Montz 2018 ^c^ [35]	US	Single center	University of Iowa Children’s Center	NR ^e^	IVA: 8	NR	0	NR
					COMP: 16			NR
Wainwright 2014 ^c^ [36], Wainwright 2014 ^c^ [37]	Australia	Multicenter (IVA), Registry (COMP)	NR (IVA), ACFDR (COMP)	12	IVA: 17	*G551D*	100	38.3 (12.4) [37]
					COMP: 314	NR		45.4 (14.5) [37]
**Studies without a non-ivacaftor-treated comparator**								
**>50 pwCF treated with ivacaftor (11 studies)**								
Bonafede 2014 ^c^ [38]	US	Administra-tive claims data	Truven Health MarketScan Commercial Database	6	102	NR	60	NR
Castellani 2018 ^c^ [39], Castellani 2018 ^c^ [40] (VOCAL)	UK, Nether-lands, Italy	Multicenter	15 sites in Italy, Netherlands, and UK (interim analysis)	12	71	Non-*G551D* gating	68	64.7 (24.5) [39]
Guimbellot 2018 ^c^ [41] (GOAL/GOAL-e2)	US	Multicenter	28 centers within the Cystic Fibrosis Therapeutics Development Network (GOAL study)	66	96	*G551D*	46	82 (NR)
Guimbellot 2019 [42]				6	21 ^f^	Non-*G551D* gating	48	68.0 (28.4)
Hathorne 2015 ^c^ [43],				12	18 ^f^	*G551D*	NR	NR
Heltshe 2015 [44]				12	151	*G551D*	54	82.6 ^g^ (25.6)
Rowe 2014 [45]				6	151	*G551D*	54	82.6 (25.6)
Sagel 2015 ^c^ [46]				6	54	*R117H*	61	85 (25)
van de Peppel 2019 [47]				6	99	*G551D*	NR	93.4 (median)
Feng 2018 [48]	US	Administra-tive claims data	Truven Health MarketScan Commercial Database	12	143	NR	63	NR
Fink 2015 ^c^ [49]	US	Registry	US CF Foundation Patient Registry (CFFPR)	12	403	*G551D*	NR	NR
Hassan 2016 ^c^ [50]	US	Administra-tive claims data	Truven MarketScan Medicaid Multi-State database	12	84	NR	56	NR
Hubert 2018 [51]	France	Registry	NR	24	57	Gating	47	72.3 (26.4)
Hubert 2018 ^c^ [52], Hubert 2018 ^c^ [53] (BRIO)	France	Multicenter	35 French CF centers (interim analysis)	12	107	Gating	48	72.6 (24.4) [53]
Kirwan 2019 [54]	Ireland	Registry	CF Registry of Ireland	36	80	*G551D*	44	71.53 (26.09)
Newsome 2018 ^c^ [55]	UK	Registry	UK CF Registry	36	361	NR	NR	NR
Suthoff 2016 [56]	US	Database	Truven Health MarketScan Commercial Database	12	79	NR	48	NR
**20–50 pwCF treated with ivacaftor (11 studies)**								
Al Redha 2016 ^c^ [57]	UAE	Single center	UAE Paediatric CF Centre	12	12	*S549R*	0	S549R/S549R, 70 (NR)
Barry 2014 [58], Barry 2015 ^c^ [59], Banerjee 2014 ^c^ [60]	UK	Single center	Manchester Adult CF Centre	12	24	*G551D*	100	64.3 (NR) [58]
Chassagnon 2016 [61]	France	Multicenter	8 French CF centers	37 (median)	22	Gating	100	39.5 (NR)
Corvol 2018 [62]	France	Multicenter	French CF Modifier Gene Study	12	30	Gating	NR	NR
Deane 2015 ^c^ [63] (CORK)	Ireland	Single center	Cork CF Centre	12	20	*G551D*	100	NR
Hickey 2015 ^c^ [64] (CORK)				20 (mean)	36		NR	NR
Ronan 2015 ^c^ [65] (CORK)				NR	24		100	NR
Ronan 2018 [66] (CORK)				12	33		61.0	75.21 (20.7)
Greenawald 2018 ^c^ [67]	US	Multicenter	Nemours CF centers	12	26	Class III-V	0	NR
Hassan 2016 ^c^ [68]	US	Administra-tive claims data	Truven MarketScan Medicaid Multi-State Database	12	44	NR	14	NR
Looi 2016 ^c^ [69]	UK	Single center	Manchester Adult CF Centre	6	30	Gating	NR	NR
McCullagh 2017 ^c^ [70]	UK	Single center	Liverpool Heart & Chest Hospital	36	22	*G551D*, *S549N*	100	83.1 (NR)
Salvatore 2019 ^c^ [71]	Italy	Multicenter	NR	12	25	Class IV-V	100	31.5 (14.5)
Stallings 2018 [72]	US	Single center	Children’s Hospital of Philadelphia	3	23	Gating	NR	86 (21)
**<20 pwCF treated with ivacaftor (28 studies)**								
Al-Rashdi 2019 ^c^ [73]	Oman	Single center	The Royal Hospital	12	15	*P.ser 549 Arg Del*	NR	54.27 (25.46)
Aziz 2016 ^c^ [74]	UK	Single center	Cambridge Centre for Lung Infection	24	15	*G551D*	100	59.6 (NR)
Carrion 2018 [75]	US	Multicenter	4 CF care centers	12	6	Class III-IV	67	49.5 (median)
Dagan 2017 [76]	Israel	Multicenter	NR	12	8	*S549R*	50	74 (23)
Ellemunter 2018 ^c^ [77]	Austria	Single center	CF Centre Innsbruck	24	7	*G551D*	NR	93.2 (NR)
Ewence 2013 ^c^ [78]	UK	Single center	Frimley Park Hospital NHS Foundation Trust	3	10	*G551D*	NR	58.2 (19.8)
Graeber 2015 [79]	Germany	Multicenter	NR	3	12	*G551D*	NR	88.8 (21.7)
Grasemann 2015 [80]	Canada	Multicenter	Hospital for Sick Children, St. Michael’s Hospital	1	15	Gating	53	69.7 (16.7)
Grasemann 2018 ^c^ [81]				24	20		65	Pediatric, 80 (NR) Adults, 65 (NR)
Green 2014 ^c^ [82]	UK	Single center	Manchester Adult CF Centre	1	13	NR	100	56.0 (NR)
Guhaniyogi 2015 ^c^ [83]	UK	Single center	All Wales Adult CF Centre	12	11	*G551D*	100	63.5 (26.2)
Hebestreit 2013 [84]	Germany	Multicenter	German CF centers	8 (mean)	14	*G551D*	100	25.0 (7.5)
Hisert 2017 [85]	Interna-tional	Multicenter	NR	24	12	*G551D*	100	64.2 (NR)
Iacotucci 2016 ^c^ [86]	Italy	Multicenter	Adult CF Center, University of Naples; CF Center, Hospital San Carlo	6	18	Non-*G551D* gating	NR	55.8 (23.6)
Jenkins 2014 ^c^ [87]	UK	Multicenter	Royal Belfast Hospital for Sick Children, Belfast Adult CF Centre	6	14	*G551D*	50	84.1 (NR)
Kane 2015 ^c^ [88]	Canada	Single center	Hospital for Sick Children	1 (median)	10	*G551D*, *G178R*	NR	70.0 (NR)
Kristensen 2016 ^c^ [89]	Multiple countries	Multicenter	HIT-CF Program	2	15	*S1251N*	NR	NR
Millar 2018 [90]	UK	Multicenter	Northern Ireland Regional Adult CF Center; Centre for Experimental Medicine, Queens University	24 (mean)	15	*G551D*	NR	NR
Mitchell 2018 ^c^ [91]	UK	Single center	Manchester Adult CF Centre	12	12	Class IV–V	NR	31.3 (5.4)
Mouzaki 2017 ^c^ [92]	Canada	Multicenter	Hospital for Sick Children, St. Michael’s Hospital	24	18	Gating	NR	76 (20)
Robson 2019 ^c^ [93]	UK	Single center	Leeds Regional Paediatric CF Centre	60	10	Gating	0	87.0 (median)
Salvatore 2018 ^c^ [94]	Italy	Multicenter	NR	6	9	Class IV-VI	100	37.3 (9.4)
Salvatore 2019 ^c^ [95]	Italy	Multicenter	Compassionate-use program	12	10	Class IV-VI	100	NR
Salvatore 2019 [96]	Italy	Multicenter	Compassionate-use program	12	13	Non-*G551D* gating	92	35.1 (14.3)
Sermet-Gaudelus 2016 [97]	France	Single center	NR	20 (mean)	7	*Gly551Asp*	100	48 (9)
Sheikh 2015 [98], Sheikh 2015 [99]	US	Single center	CF Center at Nationwide Children’s Hospital	12	12	*G551D*	50	82.5 (26) [98]
Spoletini 2019 ^c^ [100]	UK	Single center	Adult CF Unit, Leeds	24	9	*R117H*	NR	NR
Tierney 2018 ^c^ [101]	Australia	Single center	Alfred Health CF Service	24	11	*G551D*	100	61.8 (22.9)
Trinh 2013 ^c^ [102]	France	Single center	Pulmonary Department, Hopitaux Universitaires Paris Centre	11	7	*G551D*	100	44.1 (NR)

^a^ Cross-sectional analysis using 2014 data from US and UK CF registries. UK; mean ivacaftor exposure, 1.3 years and US; mean ivacaftor exposure, 2.0 years. ^b^ As ivacaftor was initially approved for use in patients aged ≥6 years with one or more gating mutations, the majority of patients in the ivacaftor-treated cohorts were expected to have a gating mutation on one of the alleles (Class III mutations) and comparator cohort predominantly included pwCF with Class I-II mutations. ^c^ Results presented in conference abstracts. ^d^ Study included 28 total patients; number of pwCF receiving ivacaftor was not reported. ^e^ Results reported as change per year. ^f^ Subgroup analysis of the GOAL study in pwCF with *G551D* and non-*G551D* gating mutations treated with ivacaftor. ^g^ As populations were identical, baseline ppFEV_1_ data captured from Rowe 2014 [45]. ACFDR: Australian Cystic Fibrosis Data Registry; CF: cystic fibrosis; CFTR: cystic fibrosis transmembrane conductance regulator; COMP: comparator; GOAL: *G551D* Observational Study; GOAL-e2: *G551D* Observational Study Expanded and Extended; IVA: ivacaftor-treated; LTSS: Long-Term Safety Study; NHS: National Health Service; NR: not reported; pwCF: people with cystic fibrosis; SD: standard deviation; UAE: United Arab Emirates.

## Data Availability

Not applicable.

## Supplementary Materials

The following are available online at https://www.mdpi.com/article/10.3390/jcm10071527/s1, Table S1: Systematic literature review search strategy, Table S2: Outcomes reported by study.
