# Supplementary material for: Real-World Outcomes of Ivacaftor Treatment in People with Cystic Fibrosis: A Systematic Review
Source: J Clin Med. 2021 Apr 6;10(7):1527. doi: 10.3390/jcm10071527 (PMC8038673; doi:10.3390/jcm10071527)
Supplement: Supplementary file 1 [file jcm-10-01527-s001.pdf]

Journal of Clinical Medicine

MDPI

Review

Real-World Outcomes of Ivacaftor Treatment in People with Cystic Fibrosis: A Systematic Review

Jamie Duckers [1,*], Beth Lesher [2], Teja Thorat [3], Eleanor Lucas [2], Lisa McGarry [3], Keval Chandarana [3] and Fosca De Iorio [4]

1 All Wales Adult Cystic Fibrosis Centre, University Hospital Llandough, Cardiff, CF64 2XX, UK
2 Pharmerit – An OPEN Health Company, 4350 East-West Highway, Suite 1100, Bethesda, MD 20814, USA
3 Vertex Pharmaceuticals Incorporated, Boston, MA 02210, USA
4 Vertex Pharmaceuticals (Europe) Limited, London, W2 6BD, UK
* Correspondence: Jamie.Duckers@wales.nhs.uk; Tel.: +44 2920 715052

Received: 3 February 2021; Accepted: 26 March 2021; Published: 6 April 2021

Supplementary Material

Table S1. Systematic literature review search strategy.

Topic	Set	String
**Cystic fibrosis**	S1	MESH.EXACT(“Cystic Fibrosis”) OR EMB.EXACT(“Cystic Fibrosis”) OR TI,AB(“Cystic Fibrosis”) OR TI,AB(“CF") OR MESH.EXACT(“Mucoviscidosis”) OR EMB.EXACT(“Mucoviscidosis”) OR TI,AB(“Mucoviscidosis”)
**Ivacaftor search**	S3	TI,AB(KALYDECO OR ivacaftor OR 873054-44-5)
**Real-world evidence search**	S4	TI,AB(“epidemiologic studies”) OR TI,AB(“case control studies”) OR TI,AB(“family study”) OR TI,AB(“longitudinal study") OR TI,AB(“retrospective study”) OR TI,AB(“retrospectively”) OR TI,AB(“prospective study”) OR TI,AB(“cohort analysis”) OR TI,AB(“cohort study”) OR TI,AB(“cohort studies”) OR TI,AB("case control study”) OR TI,AB(“follow up”) OR TI,AB("observational study”) OR TI,AB(“observational studies”) OR TI,AB("cross sectional study”) OR TI,AB(“cross sectional studies”) OR TI,AB(cohort NEAR/5 (study OR studies)) OR TI,AB(“case control” NEAR/5 (study OR studies)) OR TI,AB(“follow up” NEAR/5 (study OR studies)) OR TI,AB(observational NEAR/5 (study OR studies)) OR TI,AB(epidemiologic* NEAR/5 (study OR studies)) OR TI,AB(“cross sectional” NEAR/5 (study OR studies)) OR TI,AB(“disease registry” or “disease registries”) OR EMB.EXACT(“case control study”) OR EMB.EXACT(“family study”) OR EMB.EXACT(“longitudinal study”) OR EMB.EXACT(“retrospective study”) OR EMB.EXACT(“prospective study”) OR EMB.EXACT(“cohort analysis”) OR EMB.EXACT(“follow up”) OR EMB.EXACT(“observational study”) OR EMB.EXACT(“epidemiology”) OR EMB.EXACT(“cross-sectional study”) OR EMB.EXACT("disease registry") OR MESH.EXACT(“Case-Control Studies”) OR MESH.EXACT(“Longitudinal Studies”) OR MESH.EXACT(“Retrospective Studies”) OR MESH.EXACT(“Prospective Studies”) OR MESH.EXACT(“Cohort Studies”) OR MESH.EXACT(“Follow-Up Studies”) OR MESH(“Observational Studies”) OR MESH.EXACT(“Epidemiologic Methods”) OR MESH.EXACT(“Cross-Sectional Studies”)

Table S2. Outcomes reported by study.

**Reference (study)**	**$ppFEV_1$**	**PEx**	**Weight**	**BMI**	**Hospitalizations**	**Antibiotic Use/Duration**	***P. aeruginosa***	**CFQ-R Respiratory**	**SNOT Overall Score**	**Organ Transplant**	**Mortality**	**Other**
**Studies with non-ivacaftor-treated comparator**												
**>50 pwCF treated with ivacaftor (three studies)**												
Bell 2019 [1]								X				
Bessonova 2018 [2], Volkova 2020 [3] (LTSS)	X	X			X		X			X	X	CFRD, *S. aureus*, aspergillus, adverse events, complications
Frost 2019 [4]						X	X					*S. aureus*, aspergillus
**20–50 pwCF treated with ivacaftor (two studies)**												
Barry 2014 [5], Barry 2015[a] [6]	X			X		X				X	X	Adverse events
Emery 2019[a] [7]	X			X								Pancreatic replacement enzyme
**<20 pwCF treated with ivacaftor (two studies)**												
McLearn-Montz 2018[a] [8]												Height *z*-score
Wainwright 2014[a] [9], Wainwright 2014[a] [10]	X		X	X	X							

**Reference (study)**	**ppFEV$_1$**	**PEx**	**Weight**	**BMI**	**Hospitalizations**	**Antibiotic Use/Duration**	***P. aeruginosa***	**CFQ-R Respiratory**	**SNOT Overall Score**	**Organ Transplant**	**Mortality**	**Other**
**Studies without a non-ivacaftor-treated comparator**												
**>50 pwCF treated with ivacaftor (11 studies)**												
Bonafede 2014[a] [11]					X							
Castellani 2018[a] [12], Castellani 2018[a] [13] (VOCAL)	X	X		X	X	X				X	X	Adverse events
Guimbellot 2018[a] [14], Guimbellot 2019 [15], Hathorne 2015[a] [16], Heltshe 2015 [17], Rowe 2014 [18], Sagel 2015[a] [19], van de Peppel 2019 [20] (GOAL/GOAL-e2)	X	X	X	X	X		X	X	X			Height *z*-score, aspergillus
Feng 2018 [21]					X							
Fink 2015[a] [22]	X	X	X									
Hassan 2016[a] [23]		X			X							
Hubert 2018 [24]	X		X		X	X	X					
Hubert 2018[a] [25], Hubert 2018[a] [26] (BRIO)	X	X		X	X	X				X	X	Adverse events
Kirwan 2019 [27]	X				X	X						
Newsome 2018[a] [28]	X											
Suthoff 2016 [29]					X							
**20–50 pwCF treated with ivacaftor (11 studies)**												

**Reference (study)**	**$ppFEV_1$**	**PEx**	**Weight**	**BMI**	**Hospitalizations**	**Antibiotic Use/Duration**	***P. aeruginosa***	**CFQ-R Respiratory**	**SNOT Overall Score**	**Organ Transplant**	**Mortality**	**Other**
Al Redha 2016 [a] [30]	X											
Barry 2014 [31], Barry 2015[a] [32], Banerjee 2014[a] [33]	X		X	X								Endocrine function
Chassagnon 2016 [34]	X											
Corvol 2018 [35]	X											
Deane 2015[a] [36], Hickey 2015[a] [37], Ronan 2015[a] [38], Ronan 2018 [39] (CORK)	X	X		X				X				Endocrine function, shuttle walk test
Greenawald 2018[a] [40]	X					X						*S. aureus*
Hassan 2016[a] [41]						X						
Looi 2016[a] [42]				X								
McCullagh 2017[a] [43]							X					*S. aureus*
Salvatore 2019[a] [44]	X			X		X						Adverse events
Stallings 2018 [45]	X		X	X				X				Height *z*-score, fat-free mass
**<20 pwCF treated with ivacaftor (28 studies)**												
Al-Rashdi 2019[a] [46]	X			X	X		X					
Aziz 2016[a] [47]	X											

**Reference (study)**	**ppFEV$_1$**	**PEx**	**Weight**	**BMI**	**Hospitalizations**	**Antibiotic Use/Duration**	***P. aeruginosa***	**CFQ-R Respiratory**	**SNOT Overall Score**	**Organ Transplant**	**Mortality**	**Other**
Carrion 2018 [48]	X		X	X	X		X					Pancreatic function
Dagan 2017 [49]	X					X						
Ellemunter 2018[a] [50]	X											
Ewence 2013[a] [51]	X		X									
Graeber 2015 [52]	X			X								
Grasemann 2015 [53], Grasemann 2018[a] [54]	X											
Green 2014[a] [55]	X											
Guhaniyogi 2015[a] [56]	X			Xok		X		X				
Hebestreit 2013 [57]	X		X								X	
Hisert 2017 [58]	X											
Iacotucci 2016[a] [59]	X			X								6-minute walk test
Jenkins 2014[a] [60]	X											
Kane 2015[a] [61]	X											
Kristensen 2016[a] [62]							X					*Streptococcus, veillonella, prevotella*

**Reference (study)**	**ppFEV$_1$**	**PEx**	**Weight**	**BMI**	**Hospitalizations**	**Antibiotic Use/Duration**	***P. aeruginosa***	**CFQ-R Respiratory**	**SNOT Overall Score**	**Organ Transplant**	**Mortality**	**Other**
Millar 2018 [63]						X	X					
Mitchell 2018[a] [64]	X					X						
Mouzaki 2017[a] [65]	X		X	X								Fat-free mass
Robson 2019[a] [66]	X											
Salvatore 2018[a] [67]	X			X		X						
Salvatore 2019[a] [68]	X			X		X						
Salvatore 2019 [69]	X	X	X	X							X	
Sermet-Gaudelus 2016 [70]	X		X			X						
Sheikh 2015 [71], Sheikh 2015 [72]	X		X	X	X	X						
Spoletini 2019[a] [73]						X						
Tierney 2018[a] [74]			X	X								Fat-free mass
Trinh 2013[a] [75]	X		X			X						
**Total studies reporting**	**43**	**8**	**15**	**22**	**14**	**19**	**9**	**5**	**1**	**4**	**5**	**18**

[a]Results presented in conference abstracts. BMI: body mass index; CFQ-R: Cystic Fibrosis Questionnaire-Revised; CFRD: cystic fibrosis-related diabetes; *P. aeruginosa*: *Pseudomonas aeruginosa*; PEx: pulmonary exacerbations; ppFEV$_1$: percent predicted forced expiratory volume in 1 second; pwCF: people with CF; *S. aureus*: *Staphylococcus aureus*; SNOT: Sino-Nasal Outcome Test.

References

1. Bell, S.C.; Mainz, J.G.; MacGregor, G.; Madge, S.; Macey, J.; Fridman, M.; Suthoff, E.D.; Narayanan, S.; Kinnman, N. Patient-reported outcomes in patients with cystic fibrosis with a *G551D* mutation on ivacaftor treatment: results from a cross-sectional study. *BMC Pulm. Med.* **2019**, *19*, 146, doi: 10.1186/s12890-019-0887-6.
2. Bessonova, L.; Volkova, N.; Higgins, M.; Bengtsson, L.; Tian, S.; Simard, C.; Konstan, M.W.; Sawicki, G.S.; Sewall, A.; Nyangoma, S.; Elbert, A.; Marshall, B.C.; Bilton, D. Data from the US and UK cystic fibrosis registries support disease modification by CFTR modulation with ivacaftor. *Thorax* **2018**, *73*, 731–740, doi: 10.1136/thoraxjnl-2017-210394.
3. Volkova, N.; Moy, K.; Evans, J.; Campbell, D.; Tian, S.; Simard, C.; Higgins, M.; Konstan, M.W.; Sawicki, G.S.; Elbert, A.; Charman, S.C.; Marshall, B.C.; Bilton, D. Disease progression in patients with cystic fibrosis treated with ivacaftor: Data from national US and UK registries. *J. Cyst. Fibros.* **2020**, *19*, 68–79, doi: 10.1016/j.jcf.2019.05.015.
4. Frost, F.J.; Nazareth, D.S.; Charman, S.C.; Winstanley, C.; Walshaw, M.J. Ivacaftor is associated with reduced lung infection by key cystic fibrosis pathogens: a cohort study using national registry data. *Ann. Am. Thorac. Soc.* **2019**, *16*, 1375–1382, doi: 10.1513/AnnalsATS.201902-122OC.
5. Barry, P.J.; Plant, B.J.; Nair, A.; Bicknell, S.; Simmonds, N.J.; Bell, N.J.; Shafi, N.T.; Daniels, T.; Shelmerdine, S.; Felton, I.; Gunaratnam, C.; Jones, A.M.; Horsley, A.R. Effects of ivacaftor in patients with cystic fibrosis who carry the G551D mutation and have severe lung disease. *Chest* **2014**, *146*, 152–158, doi: 10.1378/chest.13-2397.
6. Barry, P.J.; Plant, B.J.; Simmonds, N.J.; Bicknell, S.; Bell, N.J.; Shafi, N.T.; Daniels, T.; Gunaratnam, C.; Horsley, A.; Jones, A.M. Ivacaftor decreases mortality in G551D patients with severe lung disease. *Pediatr. Pulmonol.* **2015**, *50*, 275–276, Abstract 226, doi: 10.1002/ppul.23297.
7. Emery, J.; Mullane, D.; Chroinin, M.N. The effects of ivacaftor on pancreatic function in paediatric patients with cystic fibrosis gating mutations. *Arch. Dis. Child.* **2019**, *104*, A149–150, Abstract GP284, doi: 10.1136/archdischild-2019-epa.343.
8. McLearn-Montz, A.J.; Singh, S.B.; Larson Ode, K.; Fischer, A.J. Linear growth in children receiving ivacaftor or ivacaftor-lumacaftor for cystic fibrosis. *Pediatr. Pulmonol.* **2018**, *53*, 386, Abstract 620, doi: 10.1002/ppul.24152.
9. Wainwright, C.; Bell, S.; Morton, J.; Ryan, G.; Serisier, D.; Bye, P.; Mulrennan, S.; Daley, C.; Greville, H. The effect of ivacaftor in individuals with cystic fibrosis and severe lung disease: analysis of data from the Australian named patient programme. *Respirology* **2014**, *19*, 112, Abstract TP 166, doi: 10.1111/resp.12263.
10. Wainwright, C.; Bell, S.; Morton, J.; Ryan, G.; Serisier, D.; Greville, H.; Bye, P.; Mulrennan, S.; Daley, C. The effect of ivacaftor in individuals with CF and severe lung disease. *Pediatr. Pulmonol.* **2014**, *49*, 376–377, Abstract 442, doi: 10.1002/ppul.23108.
11. Bonafede, M.M.; Limone, B.L.; Suthoff, E.D.; Cahill, J.R. Health resource utilization among patients with cystic fibrosis who initiate ivacaftor treatment. *Pediatr. Pulmonol.* **2014**, *49*, 287, Abstract 202, doi: 10.1002/ppul.23108.
12. Castellani, C.; Colombo, C.; van der Ent, C.K.; Simmonds, N.J.; Kinnman, N.; Hassan, M.; DeSouza, C.; Kaviya, A. An observational study of ivacaftor in patients with cystic fibrosis and selected non-G551D gating mutations in UK, Italy and Netherlands: healthcare resource utilization from the first interim analysis of the vocal study. *Value Health* **2018**, *21*, S255, Abstract PSY58, doi: 10.1016/j.jval.2018.04.1717.
13. Castellani, C.; Colombo, C.; van der Ent, C.K.; Simmonds, N.J.; Kinnman, N.; Hassan, M.; DeSouza, C.; Kaviya, A. Clinical effectiveness results from the first interim analysis of the VOCAL study; an observational study of ivacaftor in patients with cystic fibrosis and selected non-G551D gating mutations. *J. Cyst. Fibros.* **2018**, *17*, S10, Abstract WS05.4, doi: 10.1016/S1569-1993(18)30146-2.
14. Guimbellot, J.S.; Baines, A.; Khan, U.; Heltshe, S.; VanDalfsen, J.; Jain, M.; Rowe, S.M.; Sagel, S. Long term effects of ivacaftor in G551D patients: five year follow-up data in GOAL-e2. *Pediatr. Pulmonol.* **2018**, *53*, 232, Abstract 226, doi: 10.1002/ppul.24152.
15. Guimbellot, J.; Solomon, G.M.; Baines, A.; Heltshe, S.L.; VanDalfsen, J.; Joseloff, E.; Sagel, S.D.; Rowe, S.M. Effectiveness of ivacaftor in cystic fibrosis patients with non-*G551D* gating mutations. *J. Cyst. Fibros.* **2019**, *18*, 102–109, doi: 10.1016/j.jcf.2018.04.004.

16. Hathorne, H.; Brand, K.M.; Britton, L.J.; Jackson, T.; Heltshe, S.; Kohler, C.; Cheong, J.; Harrington, K.; Sorscher, E.J.; Rowe, S.M. The investigation of quality of life and adherence in patients with the G551D mutation receiving ivacaftor therapy. *Pediatr. Pulmonol.* **2015**, *50*, 427, Abstract 619, doi: 10.1002/ppul.23297.
17. Heltshe, S.L.; Mayer-Hamblett, N.; Burns, J.L.; Khan, U.; Baines, A.; Ramsey, B.W.; Rowe, S.M.; on behalf of the GOAL (the G551D Observation-AL) Investigators of the Cystic Fibrosis Foundation Therapeutics Development Network. *Pseudomonas aeruginosa* in cystic fibrosis patients with G551D-CFTR treated with ivacaftor. *Clin. Infect. Dis.* **2015**, *60*, 703–712, doi: 10.1093/cid/ciu944.
18. Rowe, S.M.; Heltshe, S.L.; Gonska, T.; Donaldson, S.H.; Borowitz, D.; Gelfond, D.; Sagel, S.D.; Khan, U.; Mayer-Hamblett, N.; Van Dalfsen, J.M.; Joseloff, E.; Ramsey, B.W.; on behalf of the GOAL Investigators of the Cystic Fibrosis Foundation Therapeutics Development Network. Clinical mechanism of the cystic fibrosis transmembrane conductance regulator potentiator ivacaftor in G551D-mediated cystic fibrosis. *Am. J. Respir. Crit. Care Med.* **2014**, *190*, 175–184, doi: 10.1164/rccm.201404-0703OC.
19. Sagel, S.D.; Heltshe, S.L.; Khan, U.; VanDalfsen, J.; Joseloff, E.; Rowe, S.M. Effect of ivacaftor in R117H patients following FDA approval: early results of the G551D Observational-expanded and Extended (GOAL-E2) study. *Pediatr. Pulmonol.* **2015**, *50*, 261, Abstract 190, doi: 10.1002/ppul.23297.
20. van de Peppel, I.P.; Doktorova, M.; Berkers, G.; de Jonge, H.R.; Houwen, R.H.J.; Verkade, H.J.; Jonker, J.W.; Bodewes, F.A.J.A. IVACAFTOR restores FGF19 regulated bile acid homeostasis in cystic fibrosis patients with an S1251N or a G551D gating mutation. *J. Cyst. Fibros.* **2019**, *18*, 286–293, doi: 10.1016/j.jcf.2018.09.001.
21. Feng, L.B.; Grosse, S.D.; Green, R.F.; Fink, A.K.; Sawicki, G.S. Precision medicine in action: the impact of ivacaftor on cystic fibrosis-related hospitalizations. *Health Aff. (Millwood)* **2018**, *37*, 773–779, doi: 10.1377/hlthaff.2017.1554.
22. Fink, A.; Sawicki, G.S.; Morgan, W.J.; Schechter, M.S.; Rosenfeld, M.; Marshall, B.C. Treatment response to ivacaftor in clinical practice: Analysis of the US CF foundation patient registry. *Pediatr. Pulmonol.* **2015**, *50*, 361, doi: 10.1002/ppul.23297.
23. Hassan, M.; Bonafede, M.M.; Limone, B.L.; Hodgkins, P.; Suthoff, E.D.; Sawicki, G. Reduction in pulmonary exacerbations (PEx) after initiation of ivacaftor: a retrospective cohort study among patients with cystic fibrosis (CF) treated in real-world settings. *J. Cyst. Fibros.* **2016**, *15*, S58, Abstract 28, doi: 10.1016/S1569-1993(16)30268-5.
24. Hubert, D.; Dehillotte, C.; Munck, A.; David, V.; Baek, J.; Mely, L.; Dominique, S.; Ramel, S.; Danner Boucher, I.; Lefeuvre, S.; Reynaud, Q.; Colomb-Jung, V.; Bakouboula, P.; Lemonnier, L. Retrospective observational study of French patients with cystic fibrosis and a Gly551Asp-*CFTR* mutation after 1 and 2 years of treatment with ivacaftor in a real-world setting. *J. Cyst. Fibros.* **2018**, *17*, 89–95, doi: 10.1016/j.jcf.2017.07.001.
25. Hubert, D.; Fajac, I.; Munck, A.; Marguet, C.; Benichou, J.; Payen-Champenois, C.; Jha, L.; Hassan, M.; DeSouza, C.; Kinnman, N. An observational study of ivacaftor in patients with cystic fibrosis in France: first interim analysis of health care resource utilization from the BRIO study. *Value Health* **2018**, *21*, S342, Abstract PND80, doi: 10.1016/j.jval.2018.09.2046.
26. Hubert, D.; Fajac, I.; Munck, A.; Marguet, C.; Benichou, J.; Payen-Champenois, C.; Kaviya, A.; Hassan, M.; DeSouza, C.; Kinnman, N.; BRIO Study Group VX14-770-118. Clinical effectiveness from the first interim analysis of the BRIO study: an observational study of cystic fibrosis patients treated with ivacaftor in France. *Pediatr. Pulmonol.* **2018**, *53*, 160, Abstract 34, doi: 10.1002/ppul.24152.
27. Kirwan, L.; Fletcher, G.; Harrington, M.; Jeleniewska, P.; Zhou, S.; Casserly, B.; Gallagher, C.G.; Greally, P.; Gunaratnam, C.; Herzig, M.; Linnane, B.; McElvaney, N.G.; McKone, E.F.; McNally, P.; Mullane, D.; Ní Chróinín, M.; O'Mahony, M.; Plant, B.J.; Jackson, A.D. Longitudinal trends in real-world outcomes after initiation of ivacaftor: a cohort study from the Cystic Fibrosis Registry of Ireland. *Ann. Am. Thorac. Soc.* **2019**, *16*, 209–216, doi: 10.1513/AnnalsATS.201802-149OC.
28. Newsome, S.J.; Keogh, R.H.; Daniel, R.M.; CF-EpiNet. The effects of 3-year ivacaftor use on lung function and intravenous days seen in UK CF Registry Data. *J. Cyst. Fibros.* **2018**, *17*, S54, Abstract IPD2.03, doi: 10.1016/S1569-1993(18)30287-X.
29. Suthoff, E.D.; Bonafede, M.; Limone, B.; O'Callaghan, L.; Sawicki, G.S.; Wagener, J.S. Healthcare resource utilization associated with ivacaftor use in patients with cystic fibrosis. *J. Med. Econ.* **2016**, *19*, 845–851, doi: 10.1080/13696998.2016.1178125.
30. Al Redha, K.Y.; Shebani, E.; Al Nuaimi, A.; Panickar, J. Comparison of FEV1 and BMI of the 4 common CF mutations in UAE. *J. Cyst. Fibros.* **2016**, *15*, S118, Abstract 263, doi: 10.1016/S1569-1993(16)30501-X.

31. Barry, P.J.; Jones, A.M.; Webb, A.K.; Horsley, A.R. Sweat chloride is not a useful marker of clinical response to Ivacaftor. *Thorax* **2014**, *69*, 586–587, doi: 10.1136/thoraxjnl-2013-204532.
32. Barry, P.J.; Banerjee, A.; Horsley, A.; Brennan, A.L. Impact of ivacaftor on glycaemic health in patients carrying the G551D mutation. *J. Cyst. Fibros.* **2015**, *14*, S104, Abstract 182, doi: 10.1016/S1569-1993(15)30359-3.
33. Banerjee, A.; Brennan, A.L.; Horsley, A.R.; Barry, P.J. Prospective examination of the effects of ivacaftor on glycaemic health. *Thorax* **2014**, *69*, A162, Abstract P195, doi: 10.1136/thoraxjnl-2014-206260.324.
34. Chassagnon, G.; Hubert, D.; Fajac, I.; Burgel, P.R.; Revel, M.P.; on behalf of the investigators. Long-term computed tomographic changes in cystic fibrosis patients treated with ivacaftor. *Eur. Respir. J.* **2016**, *48*, 249–252, doi: 10.1183/13993003.01918-2015.
35. Corvol, H.; Mésinèle, J.; Douksieh, I.H.; Strug, L.J.; Boëlle, P.Y.; Guillot, L. *SLC26A9* gene is associated with lung function response to ivacaftor in patients with cystic fibrosis. *Front. Pharmacol.* **2018**, *9*, 828, doi: 10.3389/fphar.2018.00828.
36. Deane, J.; Ronan, N.J.; O'Callaghan, G.P.; Fouhy, F.; Rea, M.C.; O'Sullivan, O.; Hill, C.J.; Shanahan, F.; Ross, R.P.; McCarthy, M.; Murphy, D.M.; Eustace, J.A.; Stanton, C.; Plant, B.J. Clinical outcomes of real-world Kalydeco (CORK) study – investigating the impact of CFTR potentiation on the intestinal microbiota, exocrine pancreatic function and intestinal inflammation prospectively over 12 months. *J. Cyst. Fibros.* **2015**, *14*, S29, Abstract WS16.1, doi: 10.1016/S1569-1993(15)30090-4.
37. Hickey, C.; Shanahan, P.; Ronan, N.; Shortt, C.; McCarthy, M.; Fleming, C.; Howlett, C.; Cronin, K.; O'Donovan, D.; Jennings, R.; Keating, E.; NiChroinin, M.; Mullane, D.; Murphy, D.; Plant, B.J. A retrospective analysis of patient chest physiotherapy practices and adherence to inhaled therapies before and after CFTR modulation with ivacaftor. *Pediatr. Pulmonol.* **2015**, *50*, 366, Abstract 462, doi: 10.1002/ppul.23297.
38. Ronan, G.; Ronan, N.J.; Shortt, C.; Fleming, C.; Cronin, K.; McCarthy, M.; Hickey, C.; Murphy, D.M.; Eustace, J.A.; O'Halloran, D.J.; Plant, B.J. The metabolic consequences of CFTR modulation with ivacaftor in a single adult cystic fibrosis centre cohort. *J. Cyst. Fibros.* **2015**, *14*, S90, Abstract 128, doi: 10.1016/S1569-1993(15)30305-2.
39. Ronan, N.J.; Einarsson, G.G.; Twomey, M.; Mooney, D.; Mullane, D.; NiChroinin, M.; O'Callaghan, G.; Shanahan, F.; Murphy, D.M.; O'Connor, O.J.; Shortt, C.A.; Tunney, M.M.; Eustace, J.A.; Maher, M.M.; Elborn, J.S.; Plant, B.J. CORK Study in cystic fibrosis: sustained improvements in ultra-low-dose chest CT scores after CFTR modulation with ivacaftor. *Chest* **2018**, *153*, 395–403, doi: 10.1016/j.chest.2017.10.005.
40. Greenawald, L.; Shenoy, A.; Elidemir, O.; Livingston, F.; Schaeffer, D.; Chidekel, A. Real world effectiveness of ivacaftor in pediatric cystic fibrosis patients. *Pediatr. Pulmonol.* **2018**, *53*, 157, Abstract 26, doi: 10.1002/ppul.24152.
41. Hassan, M.; Bonafede, M.M.; Limone, B.L.; Hodgkins, P. One-year evaluation of pulmonary exacerbation outcomes among patients with cystic fibrosis initiated on ivacaftor in a multistate Medicaid population. *Pediatr. Pulmonol.* **2016**, *51*, 355, Abstract 426, doi: 10.1002/ppul.23576.
42. Looi, E.; Jones, A.; Barry, P.J. Ivacaftor therapy increases BMI but does not affect serum cholesterol in patients with gating mutations. *Pediatr. Pulmonol.* **2016**, *51*, 431, Abstract 619, doi: 10.1002/ppul.23576.
43. McCullagh, M.; Wright, L.; Frost, F.; Greenwood, J.; Nazareth, D.; Walshaw, M. Long term microbiological outcomes of ivacaftor use. A single-centre retrospective study. *Pediatr. Pulmonol.* **2017**, *52*, 346, Abstract 345, doi: 10.1002/ppul.23840.
44. Salvatore, D.; Braggion, C.; Calderazzo, M.A.; Cresta, F.; Majo, F.; Messore, B.; Pisi, G.; Pizzamiglio, G.; Biglia, C.; Botti, M.; Caloiero, M.; Civati, E.; Colangelo, C.; De Gregorio, F.; Francalanci, M.; Longo, F.; Terlizzi, V. Ivacaftor treatment in patients with severe lung disease carrying CFTR mutations with residual function. *J. Cyst. Fibros.* **2019**, *18*, S128–S129, Abstract P254, doi: 10.1016/S1569-1993(19)30547-8.
45. Stallings, V.A.; Sainath, N.; Oberle, M.; Bertolaso, C.; Schall, J.I. Energy balance and mechanisms of weight gain with ivacaftor treatment of cystic fibrosis gating mutations. *J. Pediatr.* **2018**, *201*, 229–237.e4, doi: 10.1016/j.jpeds.2018.05.018.
46. Al-Rashdi, Z.; Al-Busaidi, N. The effect of ivacaftor on adult cystic fibrosis patients at the Royal Hospital in Oman. *J. Cyst. Fibros.* **2019**, *18*, S129, Abstract P256, doi: 10.1016/S1569-1993(19)30549-1.
47. Aziz, A.; Borowicz-Klementowicz, J.; Barker, H.; Johnson, C.; Shafi, N.; Haworth, C.; Floto, A.; Hill, U. Ivacaftor-as effective in clinical practice? *Eur. Respir. J.* **2016**, *48*, Abstract PA1257, doi: 10.1183/13993003.congress-2016.PA1257.

48. Carrion, A.; Borowitz, D.S.; Freedman, S.D.; Siracusa, C.M.; Goralski, J.L.; Hadjiliadis, D.; Srinivasan, S.; Stokes, D.C. Reduction of recurrence risk of pancreatitis in cystic fibrosis with ivacaftor: case series. *J. Pediatr. Gastroenterol. Nutr.* **2018**, *66*, 451–454, doi: 10.1097/MPG.0000000000001788.
49. Dagan, A.; Cohen-Cymberknoh, M.; Shteinberg, M.; Levine, H.; Vilozni, D.; Bezalel, Y.; Bar Aluma, B.E.; Sarouk, I.; Ashkenazi, M.; Lavie, M.; Tsabari, R.; Blau, H.; Kerem, E.; Bentur, L.; Efrati, O.; Livnat, G. Ivacaftor for the p.Ser549Arg (S549R) gating mutation – The Israeli experience. *Respir. Med.* **2017**, *131*, 225–228, doi: 10.1016/j.rmed.2017.08.026.
50. Ellemunter, H.; Hindinger, C.; Steinkamp, G. Long-term effects of ivacaftor in patients with G551D mutation and mild lung disease. *J. Cyst. Fibros.* **2018**, *17*, S54, Abstract IPD2.04, doi: 10.1016/S1569-1993(18)30288-1.
51. Ewence, A.E.; Eruchie, C.N.; Higton, A.M.; Ho, T.B.L. Does Ivacaftor improve objective measurements of health in patients with the G551D cystic fibrosis transmembrane conductance regulator (CFTR) protein mutation? The experience of a UK cystic fibrosis centre. *Thorax* **2013**, *68*, A120, Abstract P101, doi: 10.1136/thoraxjnl-2013-204457.251.
52. Graeber, S.Y.; Hug, M.J.; Sommerburg, O.; Hirtz, S.; Hentschel, J.; Heinzmann, A.; Dopfer, C.; Schulz, A.; Mainz, J.G.; Tümmler, B.; Mall, M.A. Intestinal current measurements detect activation of mutant CFTR in patients with cystic fibrosis with the G551D mutation treated with ivacaftor. *Am. J. Respir. Crit. Care Med.* **2015**, *192*, 1252–1255, doi: 10.1164/rccm.201507-1271LE.
53. Grasemann, H.; Gonska, T.; Avolio, J.; Klingel, M.; Tullis, E.; Ratjen, F. Effect of ivacaftor therapy on exhaled nitric oxide in patients with cystic fibrosis. *J. Cyst. Fibros.* **2015**, *14*, 727–732, doi: 10.1016/j.jcf.2015.07.001.
54. Grasemann, H.; Klingel, M.; Avolio, J.; Gonska, T.; Tullis, E.; Ratjen, F. Effect of ivacaftor therapy on nitric oxide/l-arginine metabolism in airways of patients with cystic fibrosis. *Pediatr. Pulmonol.* **2018**, *53*, 233–234, Abstract 229, doi: 10.1002/ppul.24152.
55. Green, H.D.; Barry, P.J.; Paisey, C.; Smith, A.; Flight, W.G.; Marchesi, J.; Jones, A.M.; Horsley, A.; Mahenthiralingam, E. The effect of ivacaftor therapy on the microbial diversity of cystic fibrosis lung infection. *Thorax* **2014**, *69*, A162, Abstract P196, doi: 10.1136/thoraxjnl-2014-206260.325.
56. Guhaniyogi, L.; Speight, L.; Lea-Davies, M.; Prosser, A.; Lau, D.; Ketchell, R.I.; Duckers, J. Transformational care at the All Wales Adult CF Centre (AWACFC) – the impact of ivacaftor (Kalydeco®) one year on. *J. Cyst. Fibros.* **2015**, *14*, S92, Abstract 135, doi: 10.1016/S1569-1993(15)30312-X.
57. Hebestreit, H.; Sauer-Heilborn, A.; Fischer, R.; Käding, M.; Mainz, J.G. Effects of ivacaftor on severely ill patients with cystic fibrosis carrying a G551D mutation. *J. Cyst. Fibros.* **2013**, *12*, 599–603, doi: 10.1016/j.jcf.2013.05.006.
58. Hisert, K.B.; Heltshe, S.L.; Pope, C.; Jorth, P.; Wu, X.; Edwards, R.M.; Radey, M.; Accurso, F.J.; Wolter, D.J.; Cooke, G.; Adam, R.J.; Carter, S.; Grogan, B.; Launspach, J.L.; Donnelly, S.C.; Gallagher, C.G.; Bruce, J.E.; Stoltz, D.A.; Welsh, M.J.; Hoffman, L.R.; McKone, E.F.; Singh, P.K. Restoring cystic fibrosis transmembrane conductance regulator function reduces airway bacteria and inflammation in people with cystic fibrosis and chronic lung infections. *Am. J. Respir. Crit. Care Med.* **2017**, *195*, 1617–1628, doi: 10.1164/rccm.201609-1954OC.
59. Iacotucci, P.; Salvatore, D.; Carnovale, V.; d'Ippolito, M.; Buonaurio, S.; Celardo, A.; Colangelo, C.; Smaldore, G.; Ferrara, N. Effects of ivacaftor in cystic fibrosis patients carrying a non-G551D gating mutation. *J. Cyst. Fibros.* **2016**, *15*, S59, Abstract 31, doi: 10.1016/S1569-1993(16)30271-5.
60. Jenkins, L.E.; Reid, A.; Downey, D.G.; Elborn, J.S.; Rendall, J.C. The use of LCI as an effective tool for monitoring clinical response to ivacaftor therapy in CF patients with at least one G551D-allele. *J. Cyst. Fibros.* **2014**, *13*, S40, Abstract WS20.6, doi: 10.1016/S1569-1993(14)60125-9.
61. Kane, M.; Gonska, T.; Jensen, R.; Avolio, J.; Klingel, M.; Ratjen, F. Lung clearance index response in CF patients with class III CFTR mutations. *Pediatr. Pulmonol.* **2015**, *50*, 278, Abstract 232, doi: 10.1002/ppul.23297.
62. Kristensen, M.I.; de Winter-de Groot, K.M.; Berkers, G.; de Graaf, E.; Arets, H.G.; Bogaert, D.; van der Ent, C.K. The effect of treatment with ivacaftor on the respiratory microbial composition in the upper and lower airways. *Pediatr. Pulmonol.* **2016**, *51*, 306, Abstract 300, doi: 10.1002/ppul.23576.
63. Millar, B.C.; McCaughan, J.; Rendall, J.C.; Downey, D.G.; Moore, J.E. *Pseudomonas aeruginosa* in cystic fibrosis patients with c.1652G›A (G551D)-CFTR treated with ivacaftor – changes in microbiological parameters. *J. Clin. Pharm. Ther.* **2018**, *43*, 92–100, doi: 10.1111/jcpt.12616.
64. Mitchell, R.M.; Horsley, A.R.; Jones, A.M.; Barry, P.J. Ivacaftor therapy in patients with severe baseline lung disease carrying a residual function mutation. *J. Cyst. Fibros.* **2018**, *17*, S55, Abstract IPD2.06, doi: 10.1016/S1569-1993(18)30290-X.

65. Mouzaki, M.; Avolio, J.; Griffin, K.; Tullis, D.E.; Ratjen, F.; Gonska, T. Weight increase in CF patients on Kalydeco is due to decrease in resting energy expenditure and associated with increase in adipose tissue. *Pediatr. Pulmonol.* **2017**, *52*, 448, Abstract 601, doi: 10.1002/ppul.23840.
66. Robson, E.A.; Feltbower, R.; Lee, T. Real world ivacaftor efficacy in children: five years on... *J. Cyst. Fibros.* **2019**, *18*, S128, Abstract P252, doi: 10.1016/S1569-1993(19)30545-4.
67. Salvatore, D.; Braggion, C.; Messore, B.; Pisi, G.; Tuccio, G.; Bena, C.; Colangelo, C. Effects of ivacaftor in patients with cystic fibrosis and severe lung disease carrying CFTR mutations with residual function. *J. Cyst. Fibros.* **2018**, *17*, S54–S55, Abstract IPD2.05, doi: 10.1016/S1569-1993(18)30289-3.
68. Salvatore, D.; Braggion, C.; Messore, B.; Pisi, G.; Calderazzo, M.A.; Francalanci, M.; Colangelo, C.; De Gregorio, F.; Clivati, E.; Biglia, C.; Caloiero, M. Long-term effectiveness of ivacaftor in patients with Cystic Fibrosis carrying CFTR mutations with residual function and severe lung disease. *Ital. J. Pediatr.* **2019**, *45*, 6–7, Abstract A13, doi: 10.1186/s13052-019-0631-0.
69. Salvatore, D.; Carnovale, V.; Iacotucci, P.; Braggion, C.; Castellani, C.; Cimino, G.; Colangelo, C.; Francalanci, M.; Leonetti, G.; Lucidi, V.; Manca, A.; Vitullo, P.; Ferrara, N. Effectivenesss of ivacaftor in severe cystic fibrosis patients and non-G551D gating mutations. *Pediatr. Pulmonol.* **2019**, *54*, 1398–1403, doi: 10.1002/ppul.24424.
70. Sermet-Gaudelus, I.; Delion, M.; Durieu, I.; Jacquot, J.; Hubert, D. Bone demineralization is improved by ivacaftor in patients with cystic fibrosis carrying the p.Gly551Asp mutation. *J. Cyst. Fibros.* **2016**, *15*, e67–e69, doi: 10.1016/j.jcf.2016.09.003.
71. Sheikh, S.I.; Long, F.R.; McCoy, K.S.; Johnson, T.; Ryan-Wenger, N.A.; Hayes, D., Jr. Ivacaftor improves appearance of sinus disease on computerised tomography in cystic fibrosis patients with G551D mutation. *Clin. Otolaryngol.* **2015**, *40*, 16–21, doi: 10.1111/coa.12310.
72. Sheikh, S.I.; Long, F.R.; McCoy, K.S.; Johnson, T.; Ryan-Wenger, N.A.; Hayes, D., Jr. Computed tomography correlates with improvement with ivacaftor in cystic fibrosis patients with G551D mutation. *J. Cyst. Fibros.* **2015**, *14*, 84–89, doi: 10.1016/j.jcf.2014.06.011.
73. Spoletini, G.; Shaw, N.; Wood, A.; Gillgrass, L.; Etherington, C.; Whitaker, P.; Clifton, I.; Peckham, D. Use of ivacaftor (IVA) in patients heterozygous for R117H mutation: real-life experience in a large UK adult CF centre. *J. Cyst. Fibros.* **2019**, *18*, S128, Abstract P253, doi: 10.1016/S1569-1993(19)30546-6.
74. Tierney, A.; King, S.J.; Edgeworth, D.; Williams, E.; Finlayson, F.; Keating, D.; Clark, D.; Button, B.M.; Kotsimbos, T.; Wilson, J.W. An increase in weight and fat mass observed following five months of ivacaftor treatment plateaus at 24 months in adults with G551D-related cystic fibrosis. *J. Cyst. Fibros.* **2018**, *17*, S117, Abstract P204, doi: 10.1016/S1569-1993(18)30499-5.
75. Trinh, I.; Bermudez, E.; Abbas, N.; Viguier, F.; Hubert, D.; Guérin, C.; Chast, F. Ivacaftor in adults with cystic fibrosis: one-year experience in the real world setting. *Int. J. Clin. Pharm.* **2013**, *35*, 1331, Abstract DI08, doi: 10.1007/s11096-013-9886-5.
